# Evaluation of Potential Inhibitors of Zika Virus Envelope Protein Through Molecular Docking and Molecular Dynamics Simulation

**DOI:** 10.1016/j.virusres.2025.199630

**Published:** 2025-09-11

**Authors:** Jehad Zuhair Tayyeb, Maria Karolaynne da Silva, Aamal A. Al-Mutairi, Hanan M. Alharbi, Alaa A. Khojah, Imren Bayıl, Abdullah Yahya Abdullah Alzahrani, Zsolt Tóth, Jonas Ivan Nobre Oliveira, Magdi E.A. Zaki

**Affiliations:** a Division of Clinical Biochemistry, Department of Basic Medical Sciences, College of Medicine, University of Jeddah, Jeddah 23890, Saudi Arabia; b Department of Biophysics and Pharmacology, Bioscience Center, Federal University of Rio Grande do Norte, 59064-741, Natal/RN, Brazil; c Department of Chemistry, College of Science, Imam Mohammad Ibn Saud Islamic University (IMSIU), 11623 Riyadh, Kingdom of Saudi Arabia; d Department of Pharmaceutical Sciences, College of Pharmacy, Umm Al-Qura University, Makkah 21955, Saudi Arabia; e Department of Bioinformatics and Computational Biology, Gaziantep University, Gaziantep, Turkey; f Department of Chemistry, Faculty of Science, King Khalid University, Abha 61413, Saudi Arabia; g Faculty of Wood Engineering and Creative Industries, University of Sopron, Hungary

**Keywords:** *ZIKV*, Molecular modelling, Drug-likeness, ADMET, Quantum chemical descriptors, Molecular dynamic, QM/MM calculations

## Abstract

Zika virus (ZIKV) infection remains a global health threat with no approved antivirals or vaccines to date, creating an urgent need for therapeutics targeting ZIKV. The viral envelope (E) protein is critical for host cell entry and represents a validated target for antiviral intervention. Here, we aimed to identify natural flavonoid compounds capable of inhibiting the ZIKV E protein using a dual-phase *in silico* screening strategy. First, we performed density functional theory (DFT) calculations to optimize the structures of nine candidate flavonoids and obtain quantum chemical descriptors (electronic properties); we also evaluated their drug-likeness and ADMET profiles. Second, we conducted molecular docking of these optimized flavonoids to the E protein, followed by hybrid quantum mechanics/molecular mechanics (QM/MM) refinement and 100 ns molecular dynamics (MD) simulations with principal component analysis (PCA) and MM-PBSA binding free energy calculations to assess binding interactions and complex stability. Docking identified quercetin, pinocembrin, and naringenin as the top binders, with binding energies of –8.3, –8.1, and –8.0 kcal/mol, respectively. These lead flavonoids also exhibited favorable pharmacokinetic properties, including high predicted gastrointestinal absorption, efficient clearance, and minimal toxicity risk (no carcinogenic or organ-specific alerts). Notably, pinocembrin’s complex demonstrated the greatest stability throughout a 100 ns MD simulation, maintaining a tightly bound conformation. In conclusion, quercetin, pinocembrin, and naringenin emerge as promising ZIKV E protein inhibitors with robust target engagement and favorable drug-like profiles. Their significant translational potential as antiviral candidates warrants further *in vitro* and *in vivo* studies to confirm efficacy and safety.

## Highlights

• An integrated in silico approach was used to identify flavonoid-based compounds as potential inhibitors of the Zika virus (ZIKV) envelope protein.
• Molecular docking revealed strong binding affinities for quercetin, pinocembrin, and naringenin to the ZIKV E protein active sites.
• Quantum chemical analyses and molecular dynamics simulations confirmed the stability, reactivity, and binding strength of the selected ligands.
• Pharmacokinetic and toxicity predictions showed excellent drug-likeness, with high gastrointestinal absorption and no major safety concerns.
• These findings highlight the therapeutic potential of natural flavonoids as novel antiviral agents against ZIKV, warranting further experimental validation.

## Graphical abstract

**Figure:**
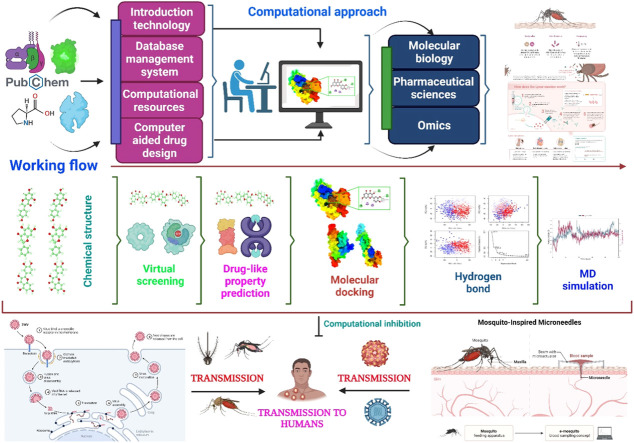

## 1 Introduction

The ongoing public health emergency caused by the mosquito-borne Zika virus (ZIKV) has elicited global concern. Historically confined to Africa and Asia, ZIKV’s emergence in Brazil in 2015 led to a global outbreak across the Americas. While the majority of ZIKV infections manifest as mild to moderate flu-like symptoms, the virus is also associated with severe complications - most notably, microcephaly in infants born to infected mothers. The absence of a definitive cure and a licensed vaccine further exacerbates the clinical challenges posed by this virus (Plourde and Bloch, 2016; Chowdhury et al., 2023; Campos et al., 2020).

The ZIKV life cycle encompasses critical phases including viral attachment, entry, translation, replication, assembly, and release. Central to these processes is the envelope protein (E protein), which plays a decisive role during the initial stages of viral entry (Gómez et al., 2022). By interacting with host cell receptors such as AXL and DC-SIGN, the E protein mediates viral binding and facilitates internalization via endocytosis (Roy et al., 2024). Once internalized, the virus is sequestered within an endosome—a vesicular compartment characterized by an acidic environment that induces conformational alterations in the E protein, ultimately triggering membrane fusion.

This fusion event is pivotal as it enables the liberation of the viral RNA genome into the host cytoplasm, thereby commandeering cellular machinery to drive replication and virion assembly. In essence, the conformational dynamics of the E protein and its role in membrane fusion make it an attractive target for antiviral drug development, since inhibiting this step could effectively prevent ZIKV infection at an early stage (Roy et al., 2024; Tréguier et al., 2022).

The primary urban vector for ZIKV transmission is the Aedes mosquito (subgenus Stegomyia), which also serves as a vector for dengue, chikungunya, and yellow fever viruses (Marcondes and Ximenes, 2015). With an estimated 3 billion individuals residing in tropical and subtropical regions at risk - due to the prevalence of Aedes aegypti, Aedes albopictus, and related species - the potential for epidemic transmission of ZIKV, dengue virus (DENV), chikungunya virus (CHIKV), and yellow fever virus (YFV) is substantial (Shragai et al., 2017; Musso and Gubler, 2016). Genomic analyses classify ZIKV strains into three principal lineages: the Nigerian cluster, the MR766 cluster, and the Asian genotype. It is hypothesized that ZIKV originated in East Africa before migrating to West Africa and subsequently to Asia. Notably, early outbreaks - such as the significant event in Yap State in 2007 - were characterized by mild clinical presentations, including rash, fever, arthralgia, and conjunctivitis, without necessitating hospitalization (Haddow et al., 2012; Retallack et al., 2016). Similar observations were reported in the Philippines (2012) and Cambodia (2010) (Alera et al., 2012; Weaver et al., 2016). Clinical data from Thailand (2012–2014) primarily documented mild cases, with fever and rash as the predominant symptoms, accompanied by sore throat, myalgia, and arthralgia, and only sporadic cases of conjunctivitis (Buathong et al., 2015).

Presently, more than 40 vaccine candidates are in various stages of development—some under accelerated approval—while one DNA vaccine has already reached phase 1 clinical trials. Nevertheless, a broadly effective vaccine remains at least two years away, and the longevity of immunity post-infection remains uncertain (Thomas et al., 2016; Cohen, 2016). In light of these challenges, this study investigates the antiviral potential of several natural compounds against ZIKV. Despite extensive *in vitro* screening demonstrating activity against various viruses, no antiviral agent has yet proven efficacious against ZIKV *in vivo*. The prevailing literature thus accentuates the imperative for effective therapeutic interventions against this pathogen (Retallack et al., 2016).

In this study, we introduce an innovative, two-phased drug design strategy aimed at targeting the ZIKV envelope protein. In the first phase, we conduct a comprehensive evaluation of selected natural flavonoid ligands. This phase involves rigorous geometry optimizations using Density Functional Theory (DFT) at the B3LYP/6–311G+ (d,p) level, detailed analysis of Molecular Electrostatic Potential (MEP) surfaces, and the determination of essential quantum chemical descriptors—including the HOMO-LUMO gap, ionization potential, and electrophilicity. Additionally, we perform extensive *in silico* assessments of drug-likeness, physicochemical properties, pharmacokinetics (ADMET), and adherence to Lipinski's rule, complemented by pharmacophore mapping analyses to elucidate key binding features.

In the second phase, the optimized ligands are integrated into a biological context through their docking into the high-resolution structure of the ZIKV envelope protein (PDB ID: 5JHM). Subsequent validation is achieved via hybrid quantum mechanics/molecular mechanics (QM/MM) calculations, followed by molecular dynamics (MD) simulations and normal mode analysis (NMA) to thoroughly investigate the dynamic stability, binding interactions, and free energy profiles of the ligand–protein complexes.

This dual-phase approach not only facilitates the identification of promising lead compounds for anti-ZIKV therapy but also provides profound mechanistic insights into the molecular interactions governing complex formation. Moreover, by integrating these advanced *in silico* techniques, our strategy minimizes reliance on animal testing and expedites the development and repurposing of effective drug candidates. Our methodology is further substantiated by recent studies (Brogi et al., 2020; Jiang et al., 2025; Huang et al., 2024; Xiong et al., 2024), which highlight the efficacy of computational approaches in modern drug discovery.

## 2 Method and material

### 2.1 Calculations of flavonoids compounds

#### 2.1.1 Ligand preparation and molecular optimization

The selection and preparation of the best ligands is one of the most critical steps in studies of potential therapeutics. This process involves identifying compounds with the highest potential for therapeutic activity and preparing them by optimizing their geometries to achieve the lowest ground-state energy. This step is essential as molecular docking and molecular dynamics (MD) simulations require ligands with well-defined geometries.

Initially, the chemical structures of flavonoid derivatives were obtained from the PubChem database in SDF format. Each structure was optimized using density functional theory (DFT) at the B3LYP/6–311G+ (d,p) level with the Gaussian 09 software (Asogwa et al., 2023). The 9 best ligands were selected for detailed analysis (Fig. 2). Moreover, the selected compounds, includes quercetin, pinocembrin, genistein, naringenin, tricin, apigenin, biochanin, taxifolin, and myricetin were based on their reported bioactivity, structural diversity among flavonoid subclasses, and pharmacological relevance as potential antiviral agents. These compounds were analyzed using DFT calculations to obtain quantum chemical descriptors, such as HOMO-LUMO. In addition, further analysis was performed using the 6–311G+ (d,p) basis set, which is one of the most commonly applied methods for ligand optimization (Rupa et al., 2022). Finally, the optimized chemical structures underwent additional computational analyses, such as molecular docking, drug-likeness, ADMET, and other relevant assessments, were conducted.

#### 2.1.2 Molecular electrostatic potential (MEP) surface

The MEP describes the combined electrostatic influence exerted by the charge distribution of a molecule at a given point in space. It includes molecular properties such as dipole moments, electronegativity, partial charges, and chemical reactivity and provides information about the relative polarity of the molecule (Murray and Sen, 1996). The MEP is a valuable tool for evaluating protein-ligand interactions involving charge differences and for predicting electrophilic and nucleophilic attacks observed in certain reactions (Karrouchi et al., 2022). In this study, the MEP surface area was calculated using the DFT/B3LYP method with a 6–311G+ (d,p) basis set.

#### 2.1.3 Quantum chemical descriptors

DFT is a computational quantum mechanical modeling method that is used extensively in various molecular optimization applications. This approach provides comprehensive insights into the behavior of electrons in molecules and their effects on crucial quantum mechanical parameters and descriptors, such as the energies of the highest occupied molecular orbital (HOMO) and the lowest unoccupied molecular orbital (LUMO), the energy gap (HOMO-LUMO), the ionization potential (I), the electron affinity (A), the chemical hardness (ɳ), the softness (σ), the chemical potential (μ), the electronegativity index (χ) and the electrophilicity index (ω) (Kawsar et al., 2022; Amin et al., 2021). The mathematical expressions (Eq. (1)) for these parameters are outlined as follows (Thanikaivelan et al., 2000):

(1) $$GAP=\varepsilon_{HOMO}-\varepsilon_{LUMO}$$

(2) $$I\approx -\varepsilon_{HOMO}$$

(3) $$A\approx -\varepsilon_{LUMO}$$

(4) σ=1/η

(5) $$\eta \approx 1/2\left(\varepsilon_{LUMO}-\varepsilon_{HOMO}\right)\approx 1/2\left(I-A\right)$$

(6) $$\mu \approx -1/2\left(\varepsilon_{HOMO}+\varepsilon_{LUMO}\right)\approx -1/2\left(I+A\right)$$

(7) χ≈−μ≈(I+A)/2

(8) $$\omega \approx \chi^{2}/2\eta$$

#### 2.1.4 Determination of ADMET, Lipinski rule, and pharmacokinetics

Predicting pharmacokinetic and toxicity characteristics of a molecule, such as its ADMET properties (absorption, distribution, metabolism, excretion, and toxicity) is an essential part of the drug development process to avoid failures in the clinical phases. Therefore, we evaluated nine selected flavonoids for their *in silico* pharmacokinetic properties to prevent them from imploding during clinical trials. The calculation of the pharmacokinetic parameters of all the natural compounds was performed via the online server pkCSM (Pires et al., 2015). This online database examines the pharmacokinetic profile of the selected compounds in terms of their water solubility, toxicity, metabolism, distribution, and absorption. On the other hand, the Lipinski rule determines the drug-like properties of any molecules. It is thought that any biomolecules that follow Lipinski rules could be an excellent oral drug. In this work, the calculation of the Lipinski rule calculation was performed by using the online web tool SwissADME (http://www.swissadme.ch/index.php) (Walters, 2012; Quantum Calculation, 2022).

#### 2.1.5 Pharmacophore mapping

PharmMapper, an online server, was used to perform pharmacophore mapping analysis for three promising ligands. The ligands were uploaded to the server in SDF format for analysis (Wang et al., 2017). The analysis included specific parameter settings to ensure accurate results. To enable a comprehensive exploration of ligand conformations, the "maximum number of conformations" was set to 1000. This allowed a thorough search for potential pharmacophore matches by expanding the conformational space. The "select target set" parameter was used to select all available targets that were chosen to cover a wide range of potential binding sites. This broad selection aimed to increase the likelihood of identifying suitable target proteins for the ligands. For further refinement, we set the "number of reserved matched targets" to 1000. This ensured that the most relevant and best-matching target proteins were prioritized in the results to focus on promising protein-ligand interactions.

In the advanced settings, a fit score cutoff value of 0 was established. The fit score represents the quality of pharmacophore matching. By selecting a cutoff value of 0, all potential matches, regardless of their fit score, were considered for further evaluation. Default settings were used for all other parameters as they were optimized within the PharmMapper server to enable reliable and consistent analyses based on established standards.

### 2.2 Calculations of flavonoid-based compound-envelope protein complexes

#### 2.2.1 Protein preparation and molecular docking study

**Fig. 1:**
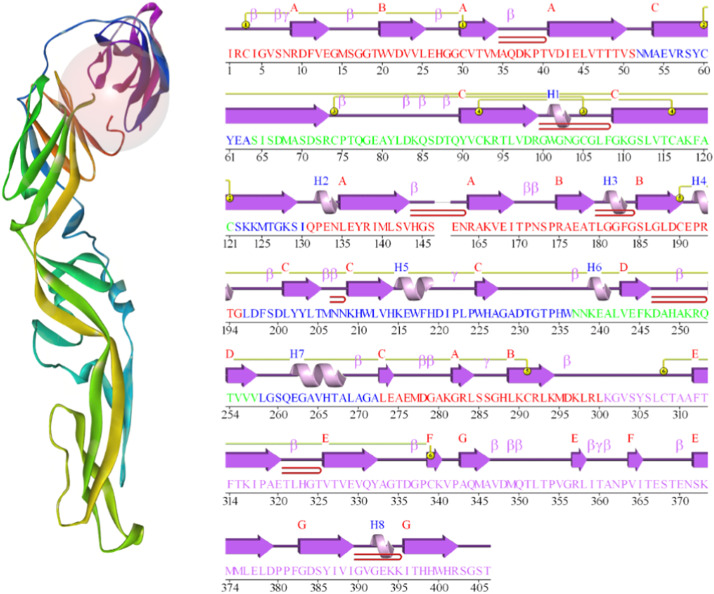
3D structural representation of the zikv envelope protein (pdb id: 5jhm) showing ligand binding site. Cartoon model highlighting the Van der Waals surface in yellow and the binding pocket in red, along with motif topology.

The three-dimensional crystal structure of the ZIKV envelope protein (PDB ID 5JHM) was downloaded from the protein data bank (https://www.rcsb.org/) (Fig. 1) (33)**.** The envelope protein’s ligand-binding pocket (highlighted in red in Fig. 1) was identified from this structure and used as the docking target. Pymol (version 1.3) software tools were used to delete all heteroatoms and water molecules (Yuan et al., 2017). This structure, resolved at 2.00 Å, which guarantees precise atomic details of the ZIKV envelope protein and thus a clear definition of the binding site. The protein was energy-minimized using Swiss PdbViewer with a force field of GROMOS96 43B1 (default parameters). Then, the targeted ZIKV protein and the previously optimized natural molecules were subjected to molecular docking using the software PyRx version 0.8 (Dallakyan and Olson, 2015). AutoDock was used to incorporate the polar hydrogens into the protein (Eberhardt et al., 2021). The grid box size on the proteins was set to (*x* = 10.04 Å, *y* = 28.65 Å, *z* = 14.114 Å), and dimension (*x* = 45.798 Å, *y* = 33.155 Å, *z* = 30.143 Å) was used. The grid coordinates were explicitly defined to encompass the ligand-binding pocket of the ZIKV envelope protein (highlighted in Fig. 1) and include all key active-site residues identified from the 5JHM crystal structure. Following the completion of the docking process, the best docked binding mode of the chemicals was complexed with the target enzyme (Dai et al., 2016; Akash, 2022). After these initial docking simulations, we performed additional docking with Dockthor, a flexible docking platform, to compare the results (Guedes et al., 2024).

#### 2.2.2 Molecular dynamics simulation (MDs), normal mode analysis (NMA)

We conducted Molecular dynamics (MD) simulations to ensure the accuracy of molecular docking, which is widely recognized as the main method for assessing the stability of protein-ligand complexes. This study provides an accurate understanding of the fluctuations and conformational changes of protein-ligand complexes (Shukla and Tripathi, 2020). In this study, MD simulations were employed to ensure the stability of quercetin, pinocembrin, and naringenin on the ZIKV envelope protein (PDB ID: 5JHM) for 100 nanoseconds (ns) using the Gromacs 2020 software package and the CHARMM36 force field within a water container with periodic boundaries. The topology files for both the protein and the ligands were generated using the CHARMM-GUI server (Che Omar, 2020; Valdés-Tresanco et al., 2021).

To maintain neutrality, a 0.15 M concentration of sodium (Na+) and chloride (Cl-) ions was added to the systems, along with three sodium ions to neutralize the charge for each of the ligands quercetin, pinocembrin, and naringenin (Streu, 2003; Ziegler, 2008). To maintain system neutrality and replicate physiological conditions, appropriate ion concentrations were added to neutralize the overall charge of the system. Energy minimization was achieved using the steepest descent method with 5000 iterations and a tolerance limit of 1000 kJ mol-1 nm-1. Under NVT conditions, the complexes were thermally equilibrated at 310 K for 125,000 picoseconds. MD simulations were performed using the NPT ensemble, which maintains a constant number of particles, pressure, and temperature. The simulations were performed for 100 nanoseconds at a temperature of 310 K, with the pressure set to 1 atmosphere and an integration time step of 2 femtoseconds. We calculated electrostatic interactions using the PME method with a Coulomb cutoff value of 1.2 nm. During the simulations, the LINCS method was used to constrain all hydrogen atom bond lengths.

After the 100-nanosecond MD simulations, the results were realigned to the center. We analyzed the trajectory data using the integrated tools in Gromacs and VMD to investigate the dynamic conformational changes and interactions within the complexes (Humphrey et al., 1996). The analyzed simulated trajectories were used to calculate various metrics, including root mean square deviation (RMSD), root mean square fluctuation (RMSF), radius of gyration (Rg), number of hydrogen bonds (H-bonds), principal component analysis (PCA), and dynamic cross-correlation matrix (DCCM)

Normal mode analysis (NMA) was performed to assess the conformational stability of the docked complex using the iMODS server, focusing on the ZIKV envelope protein in complex with naringenin, pinocembrin and quercetin. The iMODS server determines deformability data, B-factors, and eigenvalues of protein-ligand interactions to project the direction and magnitude of intrinsic motions (López-Blanco et al., 2014).

We used the coarse-grained model with C-alpha (*CA*) atoms and generated 20 normal modes to capture the dynamic range of the protein. Advanced settings included a randomized fixation approach (Fix: edge, 0.5 probability) to balance global and local movements. The elastic network model (ENM) was configured with a cutoff distance suitable to identify critical interactions, and clustering was enabled to group atoms with similar dynamics. Deformation was not applied to maintain the native conformational analysis (López-Blanco et al., 2014; Tama and Sanejouand, 2001).

The evaluation of binding free energy of ligands in proteins is a crucial parameter in molecular dynamics simulations, and the calculation of the free energy plays an important role in determining this value. In this study, we employed the MM-PBSA approach to calculate the binding free energy of the interaction between ligands and the ZIKV envelope target protein. The binding free energy (ΔG) was calculated using the equation ΔG= *G*_complex_ – G _receptor_- G _ligand_, where G-complex represents the free energy of the ligand-receptor complex, G-receptor is the free energy of the unbound receptor, and G-ligand is the free energy of the isolated ligand. These values were estimated using the MMPBSA.py module from the AMBER software package (Wang et al., 2019; Terefe and Ghosh, 2022). Frames for MM/PBSA were drawn from equilibrated windows identified by RMSD/Rg plateau criteria and analyzed by block averaging. Entropic contributions (TΔS) were not included due to their high variance under practical sampling and the expectation of partial cancellation across congeneric ligands in the same binding site. Per-residue decompositions are provided to aid interpretation.

#### 2.2.3 Quantum mechanics/molecular mechanics (QM/MM) calculations

The combined quantum mechanics/molecular mechanics (QM/MM) technique was applied to select the optimum complex (ligand-receptor) from the docking result. QM/MM optimization was performed within the framework of the ONIOM multilayer method (our own Integrated Molecular Orbital and N-layer Molecular Mechanics), which is available in Gaussian code. It is considered as the best method that ensure the accuracy initio calculations of the total energy of large complexes, like biochemical systems. Noted that these systems are divided into two or three layers. The MM layer in the present study has been implemented to the receptor, whereas the QM layer was allocated to the flavonoid derivatives. An well-known B3LYP functional and basis set 6–311G+ (d,p) were applied to analysis the electronic orbitals for the QM layer, and all amino acid residues within a radius of 6.0 Å from the centroid of the ligand were allowed to move during geometry optimization (Chow et al., 2023; Chen et al., 2022).

The QM/MM method help to break down the total energy, especially the interaction energy, into separate parts. This strategy helps scientists better understand how the protein environment—right down to particular residues—affects the system, which is especially useful when many electrostatic interactions are involved (Wang et al., 2019; Senn and Thiel, 2009).

## 3 Results and discussion

### 3.1 Optimization and energetic characterization of flavonoids compounds

**Fig. 2:**
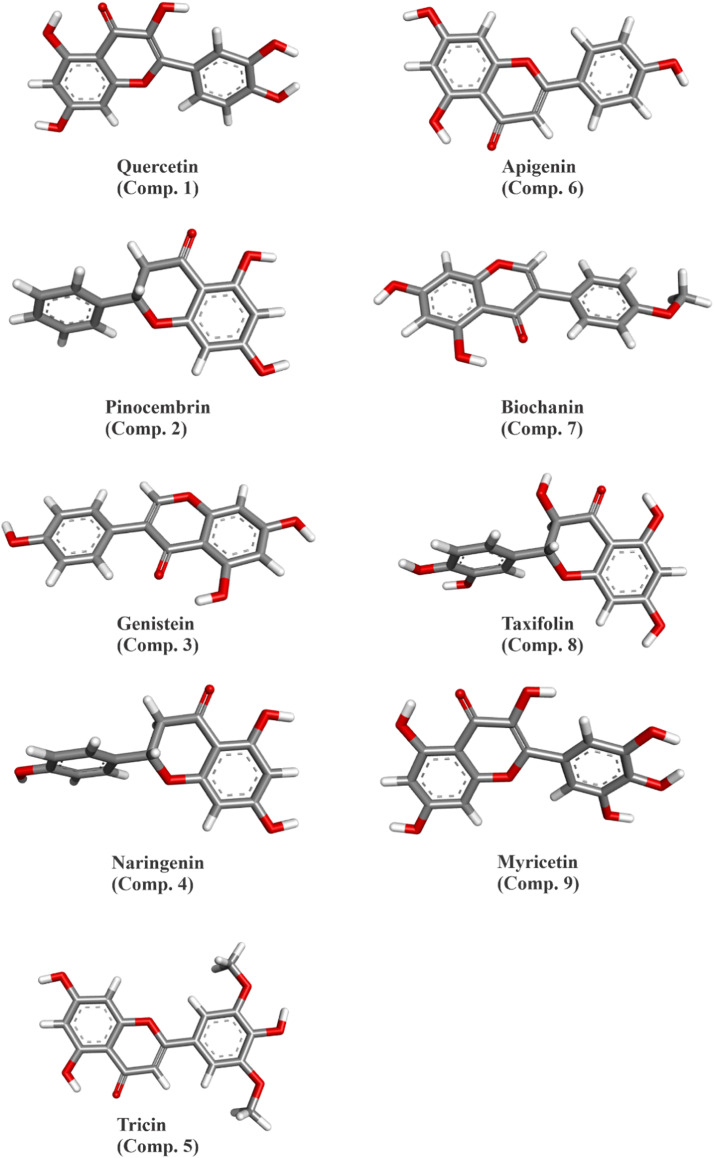
Optimized three-dimensional molecular structures of flavonoid derivatives. Geometrically optimized spatial configurations of nine flavonoids using DFT method.

In our study, nine flavonoid derivatives underwent thorough optimization and analysis (Fig. 2). Initially, the geometries were optimized with a Dreiding-like forcefield, followed by minimization with CHARMm and the SmartMinimizer approach within a distance-dependent dielectric solvent environment.

Among these compounds, Quercetin exhibited the highest number of conformations (Gómez et al., 2022), with a considerable gyrational radius (4.11 Å) and an average solvent-accessible surface area of 490.51 Å². Its pharmacophoric fingerprints totaled 1884, all of which were unique, with pairwise RMSDs ranging from 0.63 to 1.48 Å. In contrast, Pinocembrin, which has only 2 conformations, had a comparatively smaller gyrate radius (3.79 Å) and a solvent-accessible surface area (463.38 Å²). Its RMSD values remained constant at 0.17 Å, reflecting its rigidity. Similarly, Genistein also exhibited 2 conformations, with unique pharmacophoric fingerprints (813) and an average solvent-accessible surface area of 463.91 Å². The RMSD values were uniformly 0.61 Å.

Naringenin showed a higher degree of flexibility with 6 conformations. Despite its slightly smaller gyrational radius (3.77 Å), the RMSD values varied considerably, ranging from 0.09 to 1.34 Å, with an average of 0.85 Å. Tricine was characterized by the largest number of pharmacophore fingerprints (2759), all of which were unique. Its radius of gyration (4.44 Å) and solvent accessible surface area (556.39 Å²) were the highest among the derivatives analyzed. The RMSDs for tricine were very consistent, with minimal variability.

Apigenin showed similar characteristics to biochanin in terms of number of conformations (Campos et al., 2020), gyrate radius (∼4.05 Å) and solvent accessible surface area (∼467.68 Å²). Apigenin showed minimal RMSD variability, ranging from 0.09 to 0.30 Å. Biochanin exhibited unique pharmacophoric fingerprints (1293) and a high radius of gyration (4.44 Å). Its RMSD values spanned a wider range (0.48 to 0.76 Å). Taxifolin exhibited the highest flexibility with 14 conformations, an average gyrational radius of 3.68 Å and a solvent-accessible surface area of 493.95 Å². However, the RMSDs showed considerable variability with values between 0.18 and 1.94 Å. Myricetin exhibited only 2 conformations, but its average radius of gyration (4.15 Å) and solvent-accessible surface area (505.63 Å²) were relatively high. The uniformity of the RMSDs (0.13 Å) emphasizes its structural stability (Table 1).

**Table 1:** Comparative structural and physicochemical characterization of flavonoid derivatives. Summary of pharmacophore fingerprints, number of conformations, radius of gyration, solvent-accessible surface area, and RMSD values for the nine flavonoid compounds.

Compound	P.F.^1^	U.F.^2^	T.C.^3^	Avg. C./Lig.^4^	R.G.^5^ (Å)	S.A.S.A.^6^ (Å²)	Min RMSD (Å)	Max RMSD (Å)	Avg RMSD (Å)
Quercetin	1884	1884	4	4	4.11	490.51	0.63	1.48	1.15
Pinocembrin	273	273	2	2	3.79	463.38	0.17	0.17	0.17
Genistein	813	813	2	2	4.07	463.91	0.61	0.61	0.61
Naringenin	1068	1068	6	6	3.77	472.79	0.09	1.34	0.85
Tricin	2759	2759	3	3	4.44	556.39	0.5	0.52	0.5
Apigenin	688	688	3	3	4.05	467.68	0.09	0.3	0.2
Biochanin	1293	1293	3	3	4.44	495.86	0.48	0.76	0.63
Taxifolin	2402	2402	14	14	3.68	493.95	0.18	1.94	1.15
Myricetin	1588	1588	2	2	4.15	505.63	0.13	0.13	0.13

1 P.F. - Pharmacophore Fingerprints.

2 U.F. - Unique Fingerprints.

3 T.C. - Total Conformations.

4 Avg. C./Lig. - Average Conformations per Ligand;.

5 R.G. - Radius of Gyration;.

6 S.A.S.A. - Solvent Accessible Surface Area.

### 3.2 Lipinski rule and pharmacokinetics

The Lipinski rule, accessed through the SwissADME web server, was applied to further drug-likeness analysis on the reported compounds to determine the drug-like properties of our compounds. For this section and the next ones pertaining to ADMET properties, we have also included results pertaining to the FDA approved drug Ribavirin, offering a comparison to a drug currently available on the market. According to the Lipinski rule, oral molecules should have a molecular weight (M.W.) below 500 Da, no more than 5 hydrogen bond donors (HBD), no more than 10 hydrogen bond acceptors (HBA), and a logarithm of the partition coefficient (log P) below 5. Fulfilling these requirements is a prerequisite for compounds to be considered drug-like molecules (Chen et al., 2020). Based on the evaluation of all molecules, we found that their molecular weights range from 256.25 to 330.29 Dalton, their hydrogen bond acceptors range from 4 to 7, their hydrogen bond donors range from 3 to 5, and their consensus log P (o/w) ranges from 0.51 to 2.44. This current investigation shows that none of the compounds fail to meet the requirements of the Lipinski rule, indicating that all the compounds satisfy the criteria (Table 2). Likewise, Quantitative Estimates of Drug-likeness (QED) was calculated for all compounds. Pinocembrin is highlighted as the compound with highest QED, and thus the one more similar to common orally administered drugs out of all lead compounds studied. Following close behind are Biochanin and Naringenin, with a QED of 0.76 and 0.74 respectively. On the bottom 3, Myricitin, Quercetin and Taxifolin showed the lowest QED values of 0.37, 0.43 and 0.50 respectively, and as such point towards a poor oral bioavailability for these drugs. Reference drug Ribavirin presented a QED of 0.44, which would rank it as the third lowest in our analysis, further highlighting the good oral bioavailability of the other compounds.

**Table 2:** Lipinski’s Rule compliance and pharmacokinetic profiles of flavonoid candidates. Evaluation of drug-likeness based on molecular weight, hydrogen bond donors/acceptors, Log P values, and rule violations for each compound.

PubChem CID	Ligand Name	Molecular weight	Hydrogen bond acceptor	Hydrogen bond donor	Consensus Log P_o/w_	QED	Lipinski rule	
							Result	violation
5280343	Quercetin	302.34	07	05	1.23	0.43	Yes	00
68071	Pinocembrin	256.25	04	03	2.26	0.82	Yes	00
5280961	Genistein	270.24	05	03	2.04	0.63	Yes	00
439246	Naringenin	272.25	05	03	1.84	0.74	Yes	00
5281702	Tricin	330.29	07	03	2.15	0.68	Yes	00
5280443	Apigenin	270.24	05	03	2.98	0.63	Yes	00
5280373	Biochanin	284.26	05	03	2.44	0.76	Yes	00
439533	Taxifolin	304.25	07	05	0.51	0.50	Yes	00
5281672	Myricetin	318.24	08	06	0.79	0.37	Yes	01
37542	Ribavirin	244.08	09	05	−1.66	0.44	Yes	00

The Ro5 provides a useful for evaluating drug-likeness based on parameters such as molecular weight, hydrogen bond donors/acceptors, and lipophilicity. However, it is important to recognize its limitations. Many FDA-approved drugs, including antibiotics and anti-cancer agents, violate one or more Ro5 criteria, demonstrating that effective drugs can exist outside these parameters (Lohit et al., 2025). Recent studies have shown that machine learning models outperform Ro5 in predicting oral absorption, suggesting that strict adherence to Ro5 may overlook viable therapeutic candidates (Fagerholm et al., 2024).

### 3.3 ADMET data investigation

**Fig. 3:**
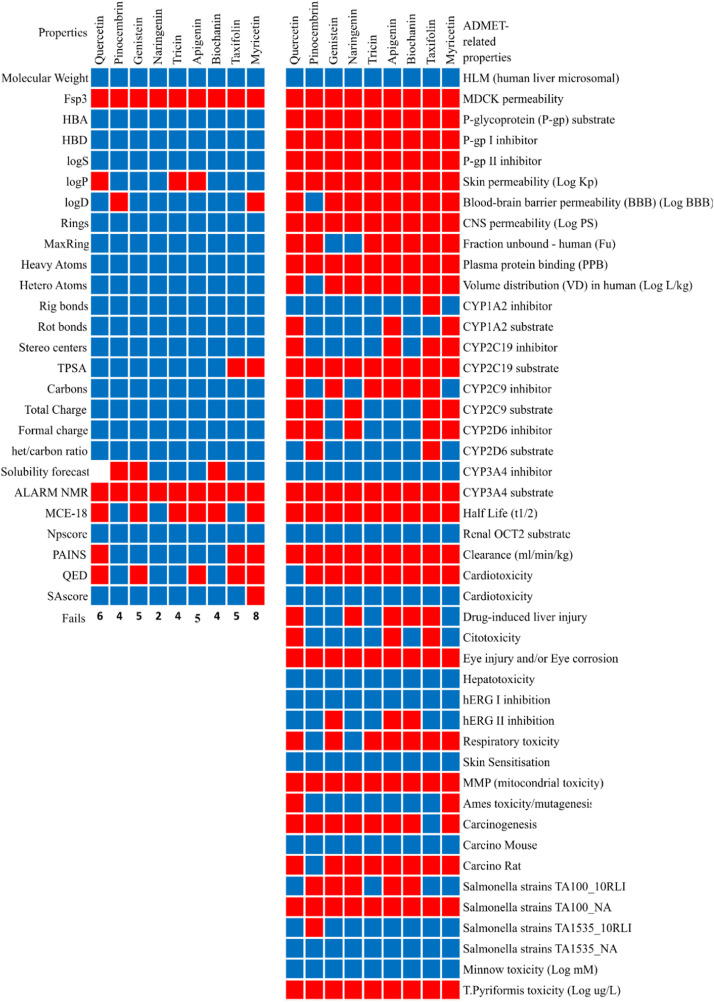
Heatmap visualization of ADMET properties across all lead flavonoid compounds. Comparative graphical display of absorption, distribution, metabolism, excretion, and toxicity data for each molecule.

**Fig. 4:**
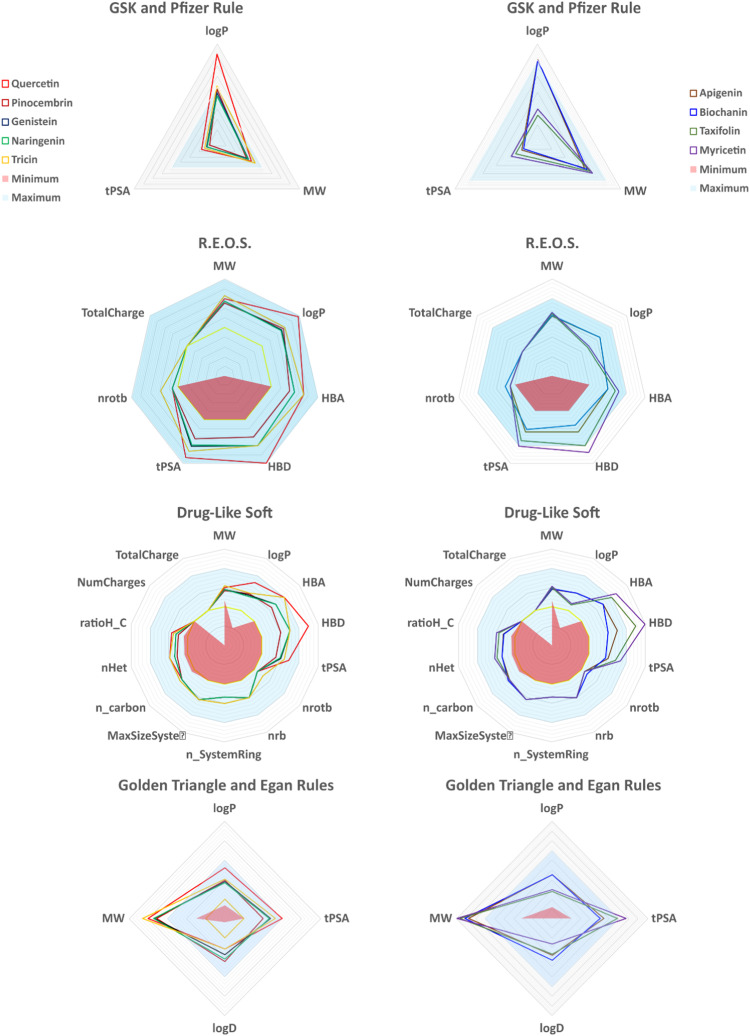
Comparative screening of physicochemical properties using multiple drug-likeness filters. Charts evaluating compliance with Pfizer, GSK, Egan, REOS, Golden Triangle, and Drug-Like Soft rules for all compounds.

The absorption, distribution, metabolism, excretion, and toxicity (ADMET) properties of the evaluated flavonoids were assessed using computational approaches. Optimal pharmacokinetic and toxicological profiles are critical for antiviral agents to ensure therapeutic efficacy while minimizing potential adverse effects (Fig. 3, Fig. 4). Quercetin and pinocembrin, for instance, show promising pharmacokinetic properties, including high gastrointestinal absorption and low predicted toxicity, aligning with the desirable characteristics of antiviral agents such as sofosbuvir and ribavirin (Kerna et al., 2024; Hussein et al., 2023; Shamim et al., 2024).

#### 3.3.1 Absorption and distribution

Effective oral bioavailability is essential for antiviral agents to achieve therapeutic concentrations in systemic circulation. Typically, the Caco-2 cell permeability assay is employed as a preliminary *in vitro* model to predict intestinal absorption. However, these assays frequently provide inconsistent or limited predictive accuracy for flavonoids, largely due to their considerable molecular weight and intricate physicochemical interactions within the gastrointestinal environment (Frenț et al., 2024; Itagaki et al., 2023). Human intestinal absorption (HIA) provides more reliable insights into systemic bioavailability (Zhang et al., 2024; Kondo and Miyake, 2023; Lin and Chiu, 2023).

Nevertheless, the limited aqueous solubility commonly exhibited by flavonoids presents substantial obstacles for achieving sufficient oral bioavailability. Specifically, quercetin, despite possessing multiple beneficial pharmacological properties, suffers from notably poor water solubility (∼1 µg/mL), significantly hampering its gastrointestinal absorption and thus restricting clinical effectiveness. This limited solubility stems primarily from its molecular structure, characterized by hydrophobic aromatic rings juxtaposed with polar hydroxyl functionalities, complicating efficient diffusion across the intestinal mucosal barrier and uptake by enterocytes (Shamim et al., 2024).

Furthermore, additional pharmacokinetic limitations of quercetin include reduced intrinsic bioactivity (<10 %), extensive metabolism (>40 %), rapid systemic clearance (plasma half-life typically <1 h), and the rapid formation of inactive metabolites (Wang et al., 2020). Clinical pharmacokinetic studies demonstrate that human absorption of quercetin ranges from approximately 3 % to 17 % of the orally administered dose, with animal models typically displaying a slightly higher absorption near 20 % (Burlou-Nagy et al., 2023). To overcome these bioavailability barriers, current biotechnological research has increasingly focused on advanced encapsulation methods and nano-delivery systems to enhance flavonoid solubility, stability, and gastrointestinal uptake, thereby maximizing their clinical applicability (Deng et al., 2019).

The predicted volume of distribution (VDss) values for the flavonoids investigated were generally low, suggesting that these compounds may have limited tissue penetration and tend to remain within the vascular compartment. While this may reduce the risk of tissue accumulation and associated toxicities, it could also necessitate more frequent dosing or formulation strategies to maintain therapeutic levels in peripheral and target tissues. On the other hand, Ribavirin exhibited a VDss of 22.12, which is on the opposite end of the other flavonoids, with values above the higher threshold of 20L/kg. Despite exhibiting high plasma protein binding (PPB > 95 %, compared to the 6.7 % of Ribavirin), which may extend plasma half-life, the limited distribution volume of the flavonoids indicates that only a small free fraction of the compound might be available for pharmacological action. These characteristics underscore the importance of optimizing delivery methods and dosage regimens to ensure adequate bioavailability and sustained therapeutic efficacy.

Furthermore, the compounds’ capability to permeate the blood-brain barrier (BBB), as demonstrated by pinocembrin, enhances their relevance in addressing neurological complications associated with neurotropic viral infections such as ZIKV (Pardridge, 2012; Kim et al., 2018).

#### 3.3.2 Metabolism and drug interactions

Flavonoid metabolism predominantly involves extensive hepatic biotransformation via cytochrome P450 (CYP) enzymes and phase II conjugation reactions. The studied flavonoids exhibited strong predicted inhibitory effects on several key CYP isoforms, notably CYP3A4 (all), CYP1A2 (8/9), CYP2C19 (5/9), and CYP2D6 (4/9), suggesting a high potential for drug-drug interactions, particularly in polypharmacy scenarios (Kondža et al., 2024; Bojić et al., 2019).

Experimental studies have confirmed that pinocembrin acts as an irreversible inhibitor of CYP3A4, reducing enzymatic activity by approximately 50 % as evaluated via testosterone 6β-hydroxylation and HPLC-DAD detection (Šarić Mustapić et al., 2018). This irreversible mechanism raises significant concerns regarding its co-administration with CYP3A4 substrate drugs, such as immunosuppressants and statins. In addition, pharmacokinetic analyses reveal that over 80 % of administered pinocembrin undergoes hepatic phase II metabolism, primarily via UDP-glucuronosyltransferases (UGTs), with negligible fecal excretion of metabolites (Amin et al., 2021). Pinocembrin strongly inhibits CYP1A2 (IC50 = 0.82 μM) and moderately inhibits CYP2C9 (IC50 = 13.1 μM) and CYP2C19 (IC50 = 22.3 μM), while exhibiting minimal inhibition of CYP2D6 and CYP3A4/5 in some settings (Amin et al., 2021). However, racemic pinocembrin has demonstrated potent inhibition of CYP2D6 at nanomolar concentrations (0.01–0.1 µM), reducing enzymatic activity by approximately 50 % (Cao et al., 2015). Furthermore, pinocembrin inhibits hepatic uptake transporters hOATP1A2 and hOATP2B1 with IC50 values of 2.0 ± 1.7 µM and 37.3 ± 1.3 µM, respectively, potentially interfering with the hepatic clearance of co-administered drugs (Sayre et al., 2013). Collectively, these findings emphasize the need for comprehensive evaluation of pinocembrin’s pharmacokinetic interactions in clinical settings.

Notably, both preclinical and clinical evidence also supports that quercetin, despite its widespread dietary consumption and natural origin, can significantly modulate drug metabolism. It has been shown to interfere with key drug-metabolizing enzymes such as CYP3A4 and efflux transporters like P-glycoprotein, leading to altered drug pharmacokinetics and bioavailability (Navrátilová et al., 2018). Ribavirin, on the other hand, showed no significant inhibitory properties or potential as a substrate for any of the CYP family enzymes analysed, but was strongly linked to OATP1B1, OATP1B3 and MRP1 inhibition.

#### 3.3.3 Excretion and clearance

Rapid excretion and efficient clearance are critical to prevent toxic accumulation and ensure pharmacokinetic adequacy. In this study, *in silico* predictions for most of the flavonoids evaluated indicated unfavorable parameters for systemic clearance and plasma half-life, suggesting rapid elimination from circulation. Such a pharmacokinetic profile may compromise therapeutic duration and efficacy. These findings underscore the need for formulation strategies aimed at enhancing metabolic stability and prolonging systemic exposure. On a positive note, none of the flavonoids were predicted to be substrates of renal OCT2 transporters, suggesting a low risk of nephrotoxicity and a favorable safety profile, particularly in patients with impaired renal function.

However, experimental data from a Phase I clinical trial presents a more favorable pharmacokinetic profile for pinocembrin than suggested by *in silico* models (Amin et al., 2021). Despite predictions of unfavorable clearance and half-life, pinocembrin demonstrated rapid hepatic metabolism and systemic elimination, with minimal accumulation upon repeated dosing. Following single intravenous administrations ranging from 20 to 150 mg, the compound exhibited linear dose-proportionality in both C_max and AUC_ (0–∞). The observed elimination half-life ranged from 40 to 55 min, and the volume of distribution (V_d) was between 137 and 175 L. Only trace amounts of unchanged parent compound were recovered in urine (<0.2 %) and feces (<2 %), confirming hepatic metabolism as the primary route of elimination, rather than renal or biliary excretion. Furthermore, no drug accumulation was observed under multiple-dose regimens, reinforcing the notion of rapid systemic clearance and highlighting the need for formulation strategies capable of sustaining therapeutic plasma concentrations.

#### 3.3.4 Detailed toxicological profiles and safety evaluations

*In silico* assessments revealed a complex and compound-specific spectrum of toxicity predictions. Among the nine flavonoids assessed, none exhibited a completely clean toxicity profile. Quercetin was predicted to be positive for cardiotoxicity, hepatotoxicity, hERG I and II inhibition, skin sensitization, AMES mutagenicity, and several carcinogenicity and microbial toxicity endpoints. Pinocembrin, despite its favorable clinical tolerability, was associated with predicted risks for cardiotoxicity, drug-induced liver injury, cytotoxicity, hERG inhibition, respiratory toxicity, skin sensitization, and multiple carcinogenic and microbial assays. Naringenin similarly showed predicted liabilities for cardiotoxicity, hepatotoxicity, hERG I and II inhibition, respiratory toxicity, skin sensitization, and potential carcinogenicity. Other flavonoids, including taxifolin, myricetin, apigenin, and biochanin, exhibited overlapping toxicological alerts, particularly concerning cardiac and hepatic endpoints, hERG inhibition, and skin sensitization. Ribavirin was not flagged as a potentially cardiotoxic in our studies, with a low likelihood of hERG inhibition; however, our analysis linked it to potential carcinogenicity, genotoxicity, and AMES mutagenicity side effects. All these potential toxicities have also been widely reported in the literature for Ribavirin.

These predicted profiles underscore the limitations of relying solely on *in silico* screening to assess safety, especially in the context of natural compounds with poly pharmacological behavior. Moreover, the presence of multiple toxicity alerts, particularly for endpoints like cardiotoxicity and hepatotoxicity, calls for prioritization of *in vitro* and *in vivo* validation assays, including cardiac ion channel profiling, liver microsome metabolism studies, and genotoxicity assessments. As such, while computational models provide an efficient early filter, they may overpredict certain toxicities or fail to reflect biotransformation and detoxification processes that modulate toxicity *in vivo*. Empirical data integration remains essential for refining the safety evaluation of flavonoid-based therapeutics.

To quercetin, human clinical studies at supplemental doses up to 1000 mg/day generally report only mild adverse events, including headaches and gastrointestinal discomfort (Andres et al., 2018). Nevertheless, preclinical studies in animal models have raised concerns about higher-dose toxicities. For example, quercetin intensified nephrotoxic effects in animals with pre-existing renal impairment and promoted tumor growth in estrogen-dependent cancer models, potentially through catechol-O-methyltransferase inhibition (Andres et al., 2018). Although initial *in vitro* assays indicated genotoxic potential, these effects have not been confirmed by subsequent *in vivo* studies at physiologically relevant doses, suggesting a low actual mutagenic risk under normal conditions. Importantly, drug interaction risks due to quercetin-mediated CYP enzyme and transporter modulation further underscore careful therapeutic monitoring (Moroy et al., 2012).

Initial Phase I clinical studies involving intravenous pinocembrin demonstrated a largely favorable safety profile. Doses up to 120 mg were well tolerated, while higher doses (150 mg) resulted in mild-to-moderate urticaria in some subjects (Cao et al., 2015). Other observed adverse events, such as Grade II diarrhea, were isolated and possibly unrelated to pinocembrin administration (Cao et al., 2015). Toxicological studies in animal models also reported no significant adverse effects or accumulation in organs following repeated administration, although enzyme inhibition (particularly irreversible inhibition of CYP3A4) suggests possible interaction risks (Šarić Mustapić et al., 2018). Additionally, pinocembrin inhibited drug transporters such as hOATP2B1 and hOATP1A2, potentially affecting the safety of co-administered drugs, notably statins (Navrátilová et al., 2018). In summary, while pinocembrin appears broadly safe in clinical and preclinical contexts, the potential for enzyme- and transporter-related adverse interactions should be carefully monitored.

Computational predictions indicated an optimal LD₅₀ and minimal toxicity for naringenin, suggesting it is safe at typical therapeutic and dietary levels. Experimental studies support this safety assessment: human clinical trials reported no clinically relevant adverse events at doses ranging from 150 to 900 mg/day (EFSA Panel on Food Additives and Flavourings (FAF) 2024). Animal toxicity assessments corroborate these findings, establishing a high NOAEL of 1320 mg/kg/day, with only subtle adverse effects—such as mild reductions in offspring growth—observed at doses far exceeding typical human exposures (EFSA Panel on Food Additives and Flavourings (FAF) 2024). Nevertheless, longer-term, multi-generational animal studies revealed subtle endocrine effects at very high chronic doses, an aspect not predicted by short-term evaluations or standard computational tools (EFSA Panel on Food Additives and Flavourings (FAF) 2024).

#### 3.3.5 Limitations of *in silico* ADMET predictions and future directions

While computational ADMET analyses provide valuable preliminary insights, significant limitations remain when assessing the toxicological profiles of flavonoids. Current predictive models are predominantly trained on synthetic drug libraries adhering to Lipinski’s rules, which inadequately capture the unique chemical features and extensive phase II conjugative metabolism (*e.g.*, glucuronidation, sulfation) characteristic of flavonoids (Frenț et al., 2024). As a result, these models often fail to accurately account for rapid biotransformation and transporter-mediated efflux mechanisms.

For example, although naringenin is frequently predicted as non-toxic, extensive *in vivo* studies have revealed subtle endocrine and immunological toxicities under high, chronic exposure conditions (EFSA Panel on Food Additives and Flavourings (FAF) 2024). Moreover, the complex interplay between flavonoids and the gut microbiome - which can significantly influence both metabolism and toxicity - is typically not captured by standard *in silico* approaches (Moroy et al., 2012).

Another critical limitation is that many *in silico* tools focus solely on the parent compound, neglecting the rapid formation of polar metabolites with distinct toxicological profiles. This oversight can lead to an overestimation of safety; for instance, quercetin’s low oral bioavailability and extensive conjugative metabolism, which contribute to its adverse effects, are not reliably predicted by generic models (Frenț et al., 2024).

Furthermore, the scarcity of high-quality experimental datasets for flavonoids hampers the refinement of computational models. Without robust pharmacokinetic and toxicological data, predictions remain largely extrapolative and subject to considerable uncertainty (Bhowmik et al., 2021). Retrospective analyses have demonstrated that exclusive reliance on *in silico* data may misjudge a flavonoid’s true safety profile, either overestimating bioavailability or overlooking potential toxic liabilities ((EFSA Panel on Food Additives and Flavourings (FAF) 2024; Bhowmik et al., 2021)).

To overcome these challenges, it is imperative to integrate comprehensive empirical validation strategies—such as targeted *in vitro* assays, rigorous *in vivo* studies, and physiologically based pharmacokinetic (PBPK) modeling—into the evaluation process. Additionally, advances in cross-docking and off-target interaction profiling across a diverse range of human proteins can further refine predictive accuracy, guiding the safer development of therapeutic agents.

Future research should prioritize *in vivo* assessments, dose optimization, and innovative formulation strategies to enhance the toxicological safety of flavonoids. Given their complex interactions with metabolic enzymes, transporters, and the gut microbiome, rigorous clinical investigations and enhanced monitoring protocols are essential. Exploring synergistic effects with existing antiviral agents may also pave the way for novel combination therapies, ultimately improving both efficacy and safety in clinical applications.

### 3.4 Quantum calculations of flavonoids compounds

#### 3.4.1 Energetic characterization

This study thoroughly analyzed several energy-related characteristics for a series of nine flavonoid compounds (Table 3). The total energy values for flavonoid base compounds have been reported ranging from −932.4411 Ha to −1248.3522 Ha, highlighting the energy of the system in its present electronic form. The observed binding energies ranged between −7.1790 Ha and −78.5988 Ha, indicating the energy released during the synthesis of each molecule. The dipole moments of the compounds ranged from 1.0806 to 3.0066 Debye, indicating charge separation inside molecules.

**Table 3:** Quantum mechanical energy parameters of optimized flavonoids. Calculated total energy, binding energy, dipole moment, solvation energy, molecular surface area, and cavity volume using DFT-based quantum method.

Name	Total energy (Ha)	Binding energy (Ha)	Dipole Mag	Dielectric sol energy (Ha)	Solvation energy (Ha)	Surface Area	Cavity volume
Quercetin	−1169.082	−74.22517	2.8120539	0.066328	0.0663282	1016.323	1977.63
Pinocembrin	−932.4411	−61.29858	1.6979104	0.046101	0.0461008	947.1669	1836.59
Genistein	−1010.535	−65.47291	2.604126	0.056329	0.056329	959.636	1864.36
Naringenin	−1011.715	−65.67548	2.1939691	0.055877	0.0558765	976.543	1889.85
Tricin	−1181.511	−7.17904	1.0805842	0.043415	0.0434149	1152.701	2245.979
Apigenin	−1010.546	−65.48369	2.808361	0.056454	0.0564537	962.9695	1864.34
Biochanin	−1052.248	−68.49523	2.4094422	0.049625	0.0496245	1030.801	2001.07
Taxifolin	−1170.251	−74.41805	3.0066148	0.068344	0.068344	1033.513	2018.95
Myricetin	−1248.352	−78.59883	2.5985562	0.06629	0.0662896	1050.55	2051.97

The dielectric solvation energy represents the polarization energy in a dielectric media, whereas the solvation energy refers to the energy shift that occurs when a molecule is transferred from a vacuum to a solvent. These parameters were determined for each molecule, and the results ranged from 0.0434 Ha to 0.0683 Ha Surface areas of the molecules ranged from 947.1669 Å² to 1152.7014 Å², indicating their interaction with the environment. Finally, cavity volumes were estimated to measure the space within molecular structures, and they ranged from 1836.59 cubic angstroms to 2245.979 cubic angstroms.

#### 3.4.2 Quantum chemical descriptors

Quantum chemical calculations are extensively utilized in chemistry and related fields, enabling the development of new materials, exploration of reaction mechanisms, and prediction of spectroscopic properties. These calculations were effectively applied in a DFT study to suggest new pharmacological possibilities (Amin et al., 2021; Kawsar et al., 2024). Quantum chemical properties such as HOMO, LUMO, GAP, ionization potential (I), electron affinity (A), and related properties like chemical hardness, softness, chemical potential, electronegativity, and electrophilicity are essential in understanding various phenomena in biology and materials science. These methods account for atomic interactions within molecules, determining electronic structure, geometry, and energy levels in both ground and excited states, which is particularly significant in drug development and molecular interaction studies (Quantum Calculation, 2022; Kawsar et al., 2024).

**Fig. 5:**
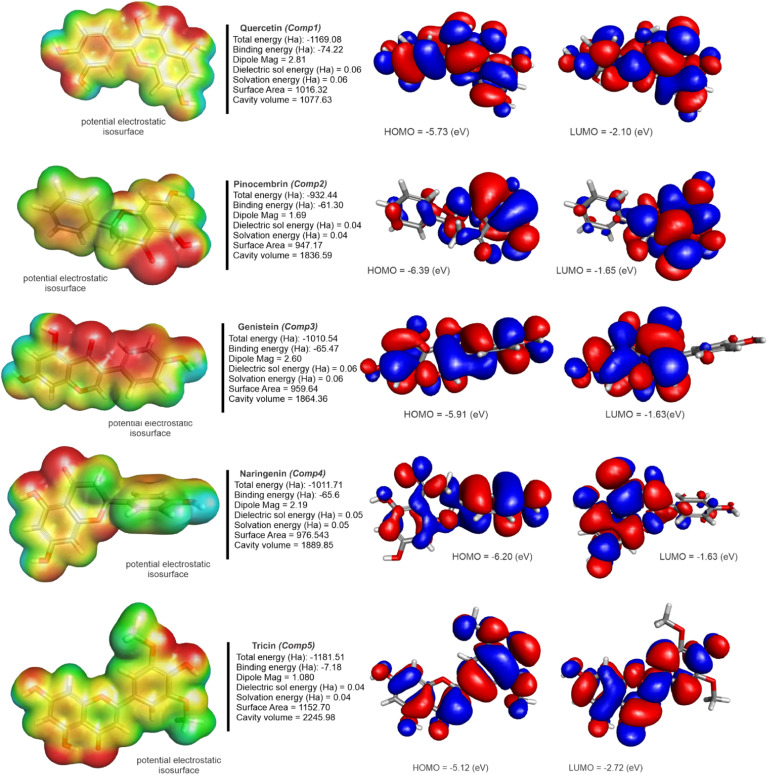
Visualization of HOMO and LUMO orbitals for Quercetin, Pinocembrin, Genistein, Naringenin, and Tricin. Frontier molecular orbital maps indicating electron donor and acceptor regions that influence reactivity and interactions.

We can determine the interaction between molecules by analyzing their frontier molecular orbitals (FMOs). This analysis involves two main orbitals: the HOMO, which is the outermost orbital that contains electrons and typically acts as an electron donor, and the LUMO, which is the innermost orbital with available space to accept electrons (Kawsar et al., 2024). Figs. 5 **and** 6 shows the frontier orbitals (HOMO and LUMO) calculated with B3LYP-6–31G+ (d,p). The energy of each HOMO (I) - LUMO (A) orbital is described in Table 4**.** The energy gap (EI, Table 4), the difference between these energies, significantly influences structural stability. Lower energy gaps are associated with more polarized and soft molecules, while higher energy gaps indicate harder molecules. Once again, the quantum chemical descriptors for Ribavirin were calculated to serve as a comparison molecule between our proposed ligands and a FDA approved drug.

**Fig. 6:**
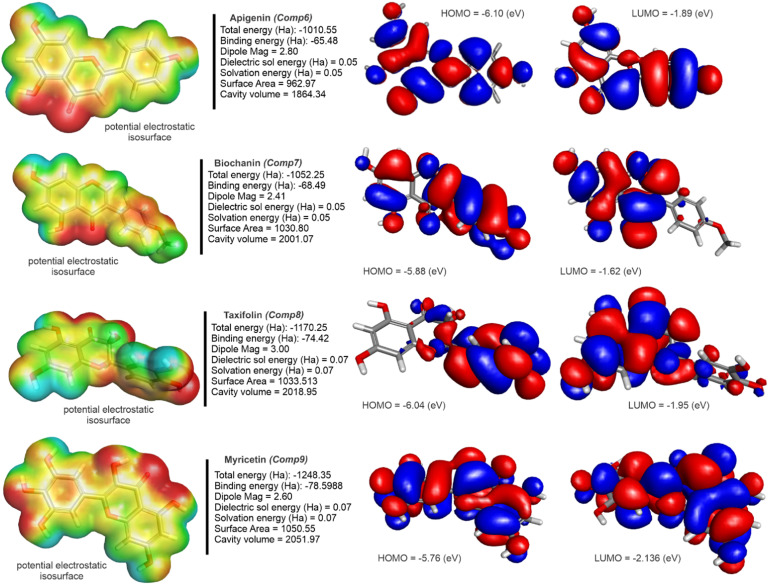
Frontier Molecular Orbitals (HOMO and LUMO) and Electrostatic Potential Isosurfaces of Apigenin, Biochanin, Taxifolin, and Myricetin.

**Table 4:** Frontier Molecular Orbital Energies and Quantum Chemical Descriptors of Flavonoid Derivatives.

Ligand	Quantum chemical descriptors *, ⁎⁎									
	HOMO	LUMO	GAP	I	A	η	σ	μ	χ	ω
Quercetin	−5.72828	−2.10629	3.62198	5.72828	2.106295	1.810992	0.552183	3.917287	−3.91729	13.89497
Pinocembrin	−6.38885	−1.65222	4.73662	6.388846	1.652222	2.368312	0.422242	4.020534	−4.02053	19.14152
Genistein	−5.91027	−1.62657	4.2837	5.91027	1.626569	2.14185	0.466886	3.76842	−3.76842	15.20819
Naringenin	−6.20836	−1.63718	4.57118	6.208361	1.637176	2.285592	0.437523	3.922769	−3.92277	17.58548
Tricin	−5.12416	−2.71963	2.40453	5.124159	2.719632	1.202263	0.831764	3.921895	−3.9219	9.246166
Apigenin	−6.10497	−1.88651	4.21846	6.104968	1.886507	2.109231	0.474107	3.995737	−3.99574	16.8379
Biochanin	−5.87005	−1.61803	4.25202	5.870048	1.618027	2.12601	0.470365	3.744038	−3.74404	14.90101
Taxifolin	−6.04355	−1.94767	4.09588	6.043548	1.947673	2.047938	0.488296	3.995611	−3.99561	16.34756
Myricetin	−5.76204	−2.13597	3.62608	5.762044	2.135966	1.813039	0.55156	3.949005	−3.949	14.13684
Ribavirin	−7.78654	−1.47268	6.31386	7.786542	1.472681	3.156931	0.316763	−4.62961	4.62961	33.83172

⁎ Ionization potential (I), electron affinity (A), chemical hardness (ɳ), softness (σ), chemical potential (μ), electronegativity index (χ) and electrophilicity index (ω);.

⁎⁎ Unit of measurement: eV.

Quercetin has a HOMO value of −5.72828 eV and a LUMO value of −2.10629 eV, resulting in a relatively low energy gap (GAP) of 3.62198 eV. This lower GAP indicates that Quercetin has a higher reactivity, which may enhance its ability to interact with biological targets.

Pinocembrin, on the other hand, shows the second most negative HOMO (−6.38885 eV) and a LUMO of −1.65222 eV, leading to the second largest energy gap among the compounds at 4.73662 eV, suggesting it is less reactive but potentially more stable. Genistein and Naringenin also exhibit relatively large energy gaps (4.2837 eV and 4.57118 eV, respectively), indicating moderate reactivity. Tricin shows the smallest GAP (2.40453 eV), suggesting it might be the most reactive of all the ligands, which could be advantageous or disadvantageous depending on the target interaction context. Ribavirin showed the highest GAP out of all compounds (6.31386 eV), hinting at its experimentally proven chemical stability.

For ionization potential (I) and electron affinity (A), quercetin presents a relatively moderate ionization potential (5.72828 eV) and electron affinity (2.106295 eV), which balances its reactivity and stability. Pinocembrin and naringenin have higher ionization potentials (6.388846 eV and 6.208361 eV, respectively), indicating that they require more energy to remove an electron, correlating with their larger energy gaps and suggesting higher stability. Tricin exhibits a lower ionization potential (5.124159 eV) and higher electron affinity (2.719632 eV), further indicating its higher reactivity compared to other ligands. At 7.786542 and 1.472681 eV for ionization and electron affinity respectively, Ribavirin once again presents itself as the more stable compound of all ligands, also correlating to the large HOMO-LUMO gap observed.

In another analysis about chemical hardness (η) and softness (σ), pinocembrin shows the second highest chemical hardness (2.368312 eV), corresponding to the lowest softness (0.422242 eV), reinforcing its stability and lower reactivity. In contrast, tricin has the lowest hardness (1.202263 eV) and the second highest softness (0.831764 eV), aligning with its high reactivity. Quercetin and myricetin demonstrate moderate values for hardness and softness, balancing between stability and reactivity. With a chemical hardness of 3.156931 and softness of 0.316763 eV respectively, Ribavirin has the highest η and lowest σ of all compounds.

For the chemical potential (μ) and electronegativity (χ), quercetin and naringenin have chemical potentials of −3.917287 eV and −3.922769 eV, respectively, with electronegativity values around −3.92 eV. These values indicate a moderate tendency to attract electrons, which is beneficial in binding interactions. Pinocembrin exhibits a slightly higher chemical potential (−4.020534 eV), making it slightly less reactive in comparison. Ribavirin presented values of −4.62961 eV for chemical potential and 4.629612 eV for electronegativity. Tricin shows a lower chemical potential (−3.921895 eV), consistent with its higher reactivity.

Latelly, electrophilicity index (ω) pinocembrin has the highest electrophilicity index (19.14152 eV) out of the proposed compounds, suggesting it is the most likely to accept electrons during a reaction, which can be advantageous in specific drug-target interactions. Quercetin and naringenin show moderately high electrophilicity values (13.89497 eV and 17.58548 eV, respectively), indicating their suitability as electrophiles in biological systems. Tricin exhibits the lowest electrophilicity (9.246166 eV), which aligns with its overall higher reactivity and less selective electron-accepting behavior. At 33.83172 eV for its electrophilicity index, Ribavirin was shown as an outlier and highlights its position as a market-available drug compared to other lead compounds.

Studies have shown that flavonoids, with their lower energy gap and high electrophilicity, effectively inhibit the replication of ZIKV by interacting with viral proteases and altering viral RNA synthesis (Cataneo et al., 2021). In addition, it shows significant antiviral activity, especially in inhibiting ZIKV post-entry processes, consistent with its stable interaction profile suggested by its quantum descriptors, particularly pinocembrin.

#### 3.4.3 Molecular electrostatic potential (MEP) surface result analysis

**Fig. 7:**
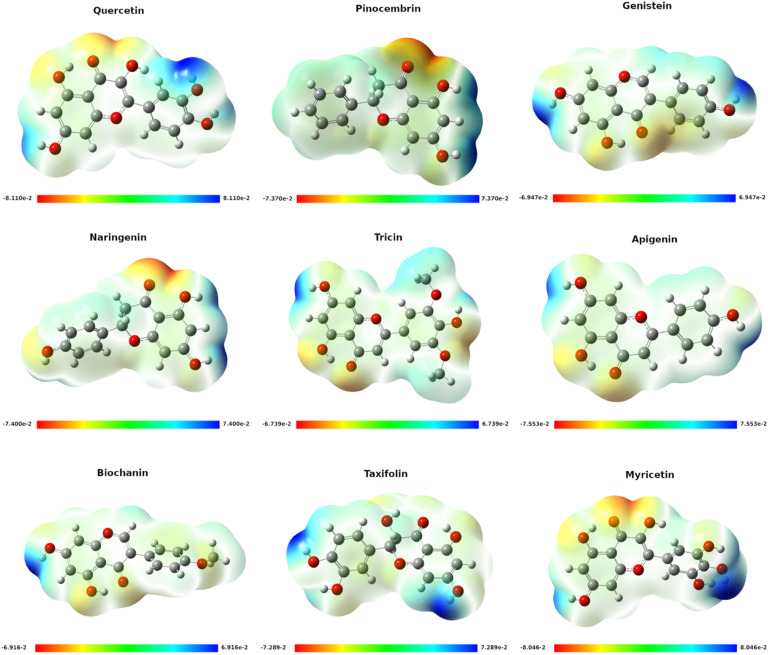
Molecular electrostatic potential (mep) surface maps and optimized structures. Electrostatic potential distributions mapped onto the molecular surface, showing electrophilic and nucleophilic regions.

Fig. 7 presents the optimized geometries of the nine flavonoid derivatives alongside their corresponding Molecular Electrostatic Potential (MEP) surfaces. This visualization offers critical insights into each molecule’s charge distribution and highlights potential sites for intermolecular interactions. In the color scale used, red regions signify higher electron density (electron-rich or nucleophilic zones), whereas blue regions denote lower electron density (electron-deficient or electrophilic zones), and green regions represent an intermediate or neutral potential.

Among these flavonoids, the oxygen-containing functional groups - particularly hydroxyl moieties - often appear in red, indicating a propensity to donate electron density or form hydrogen bonds. In contrast, hydrogen atoms linked to carbon skeletons typically emerge in blue, reflecting electron-poor areas that may engage in electrostatic attractions with negatively charged sites. By mapping these electrostatic potentials, it becomes clearer how each compound could interact with biological targets, for instance, through hydrogen bonding, π–π stacking, or other non-covalent interactions (details in). Ultimately, the MEP data assist in rationalizing both the reactivity profiles of the flavonoids and their potential binding modes to target proteins, including the ZIKV envelope protein.

### 3.5 Pharmacophore mapping

**Fig. 8:**
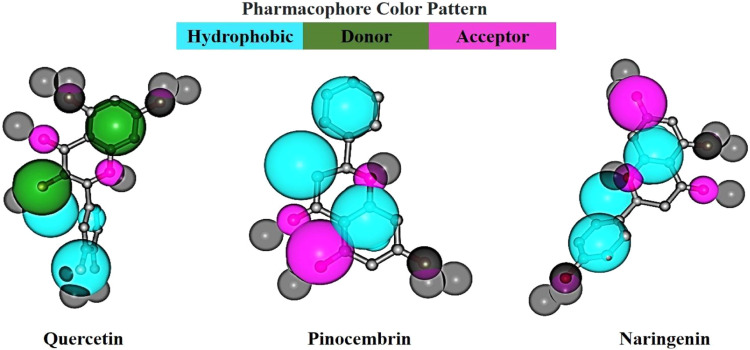
Pharmacophore mapping models of the top three compounds using PharmMapper.

In ligand-based drug design, pharmacophore mapping helps identify the essential features or chemical properties (such as hydrogen bond donors/acceptors, hydrophobic regions, aromatic rings, etc.) that are critical for a molecule to interact with a target receptor or enzyme. By incorporating the necessary pharmacophoric elements, we can use this information to design new drug candidates or optimize existing ones. In pharmacophore mapping, we use the fit score as a quantitative measure to evaluate the degree of similarity or match between a pharmacophore model and a specific chemical structure or molecule. Fig. 8 illustrates the pharmacophore mapping process. These shared substructures among the flavonoids facilitated their alignment and contributed to generating the pharmacophore model.

While the flavonoids studied are not directly known to inhibit the target protein, their fit scores, as shown in Table 6, suggest a strong correspondence with the pharmacophore model. The fit scores obtained for pinocembrin and naringenin were nearly identical, with values of 3.068 and 3.026, respectively, while the fit score for quercetin was 2.97. These relatively high fit scores indicate a strong correspondence between the pharmacophore model and the compounds, suggesting that the compounds are likely to have similar biological activity to the reference molecule or target.

The normalized fit score also gives a consistent way to measure how similar two things are, which lets researchers compare different compounds and pharmacophore models in pharmacophore mapping studies. This approach enhances our understanding of the potential biological activity and therapeutic relevance of the compounds (Gece, 2008; Mahmud et al., 2023).

In our study, we made an intriguing discovery regarding the compound's quercetin, pinocembrin, and naringenin. Interestingly, all three compounds exhibited comparable normalized fit scores, namely 0.7425, 0.767, and 0.7564, respectively (Table 5). The normalized fit score is a value that ranges from 0 to 1, with a score of 1 indicating perfect alignment between the compound and the pharmacophore model. These results suggest that all three compounds showed strong and similar matches with the pharmacophore model.

**Table 5:** Pharmacophore mapping output for top-ranked ligands (quercetin, pinocembrin, and naringenin): fit scores, normalized fit scores, and number of pharmacophoric features identified, including hydrogen bond donors, hydrogen bond acceptors, and hydrophobic centers.

Parameters	Quercetin	Pinocembrin	Naringenin
Fit score	2.97	3.068	3.026
Normalized fit score	0.7425	0.767	0.7564
Hydrophobic centre	2	3	3
Positively charged centre	0	0	0
Negatively charged centre	0	0	0
H bond donor	2	0	0
H bond acceptor	0	1	1
Aromatic ring	0	0	0

Furthermore, we examined the specific characteristics of these compounds and found that both pinocembrin and naringenin ligands possessed the same number of hydrophobic centers and hydrogen bond acceptors. Conversely, only quercetin exhibited a hydrogen bond donor, while none of the compounds had positively charged centers, negatively charged centers, or aromatic rings (as shown in Table 5). These details provide valuable insights into the structural features and functional groups present in the compounds, aiding in the understanding of their potential interactions and activity.

Overall, our study shows that quercetin, pinocembrin, and naringenin not only have similar normalized fit scores, which means they strongly agree with the pharmacophore model, but they also have unique properties that may affect how they interact with the target receptor or enzyme.

### 3.6 Molecular docking analysis of *targeted receptor of* ZIKV

With the help of molecular modeling methods, it is possible to determine the binding capacity of small molecules to target proteins. The docking process can be described in two main steps: the prediction of the ligand conformation and its position and orientation within the binding site (often referred to as the pose) and the estimation of binding affinity using a scoring function (Najmi et al., 2022; Kumer, 2022).

Using ribavirin as a reference control underscores the apparent preference of the ZIKV envelope protein (ZIKV_E) pocket for polyphenolic scaffolds. Across our panel, docking scores ranged from –6.5 to –8.3 kcal/mol, with quercetin (–8.3 kcal/mol), pinocembrin (–8.1 kcal/mol), and naringenin (–8.0 kcal/mol) ranking highest and outperforming ribavirin (–5.9 kcal/mol) by ∼2.1–2.4 kcal/mol (Table 6). This gap suggests more favorable shape and interaction complementarity for the flavonoids, which in our models can engage both polar anchors and a contiguous hydrophobic patch, supporting π-stacking/CH–π and van der Waals contacts, whereas ribavirin’s compact, highly polar nucleoside-like framework provides limited apolar surface area and is predicted to remain more solvent-exposed near the pocket entrance. Accordingly, the natural compounds are predicted to achieve tighter recognition of ZIKV_E than ribavirin; nonetheless, docking scores are approximate surrogates of affinity, and these hypotheses require biophysical and cellular validation (*e.g.*, binding assays and entry/replication readouts) to confirm potential inhibitory activity.

**Table 6:** Molecular docking binding affinity of flavonoids against ZIKV envelope Protein (PDB ID: 5JHM). Binding energies (kcal/mol) of each flavonoid compound when docked with the ZIKV envelope protein, indicating inhibitory potential.

Name of compound	Molecular Formula	ZIKV Envelope protein (PDB ID 5JHM)
		Binding Affinity (kcal/mol)
Quercetin	C_15_H_10_O_7_	−8.3
Pinocembrin	C_15_H_12_O_4_	−8.1
Genistein	C_15_H_10_O_5_	−7.7
Naringenin	C_15_H_12_O_5_	−8.0
Tricin	C_6_H_13_NO_5_	−7.4
Apigenin	C_15_H_10_O_5_	−7.0
Biochanin A	C_16_H_1_2O_5_	−6.5
Taxifolin	C_15_H_12_O_7_	−7.9
Myricetin	C_15_H_10_O_8_	−7.5
Ribavirin	C_8_H1_2_N_4_O_5_	−5.9

Upon additional docking using Dockthor, the binding affinities were further compared. Quercetin demonstrated the strongest binding affinity (−8.634 kcal/mol), followed by pinocembrin (−8.396 kcal/mol) and naringenin (−7.125 kcal/mol). These updated results support the original findings and confirm that quercetin and pinocembrin show very stable interactions with the ZIKV_E, while naringenin shows comparatively weaker binding. Furthermore, they emphasize that flavonoid-based molecules can effectively interact with the ZIKV_E, potentially stabilizing the protein in a non-functional conformation and preventing viral entry.

The strong binding affinities observed for quercetin in this study are consistent with prior reports of its antiviral activity against ZIKV. Experimental studies have shown that quercetin inhibits ZIKV replication and viral particle production in A549 and Vero cells (Cataneo et al., 2021; Saivish et al., 2023), supporting its effectiveness as a therapeutic candidate.

The high binding affinities can be attributed to specific molecular interactions such as hydrogen bonding, hydrophobic interactions, and π-π stacking, which are critical for stable ligand-protein complexes (Ozawa et al., 2017; Seo et al., 2021). Furthermore, the binding efficiency of these compounds indicates their potential to actively inhibit the ZIKV_E by stabilizing key residues within the active site, potentially disrupting the protein's function. This finding highlights the promising therapeutic potential of these flavonoids, especially quercetin and pinocembrin, against ZIKV infections (Table 6). These results emphasize the significance of natural compounds as viable candidates in the development of antiviral therapies, paving the way for further experimental and clinical validation (Pereira et al., 2023).

Molecular interaction analysis of naringenin-ZIKV_E complex, the ligand forms significant hydrogen bonds with HIS A:144 and VAL A:300, which play a central role in anchoring to the active site. These interactions are supported by hydrophobic contacts with residues such as VAL A:364, ALA A:361 and TYR A:305 as well as weak van der Waals interactions with nearby residues. Taken together, these interactions provide stable positioning of naringenin within the binding pocket and emphasize its potential as a potent inhibitor.

In the quercetin-ZIKV_E complex, a combination of hydrogen bonding and hydrophobic interactions enhances binding. Quercetin forms hydrogen bonds with ASN A:362, ASP A:37 and LYS A:39, anchoring the molecule firmly in the active site. Stabilization is enhanced by hydrophobic interactions with residues such as VAL A:356 and ALA A:361, while additional van der Waals forces involve residues such as GLN A:344 and MET A:345. This series of interactions gives quercetin a robust and stable binding orientation, supporting its potential as a potent inhibitor.

For the pinocembrin-ZIKV_E complex, a crucial hydrogen bond with HIS A:144 plays an important role in stabilizing the ligand in the active site. Hydrophobic interactions with residues such as VAL A:364 and LEU A:300 further strengthen its positioning, while weak van der Waals contacts with ASN A:362, PRO A:39 and TYR A:305 contribute to additional stabilization. These combined interactions emphasize the strong binding affinity of pinocembrin and its potential efficacy as an inhibitor.

### 3.7 Molecular dynamics simulation (MDs) and quantum mechanics/molecular mechanics (QM/MM) results

#### 3.7.1 Root mean square deviation analysis (RMSD)

Root mean square deviation (RMSD) calculations were conducted on the ligands and complexes throughout a 100 ns molecular dynamics (MD) simulation for each protein-ligand interaction to determine the structural stability, with lower values indicating greater stability (Lindorff-Larsen et al., 2010).

**Fig. 9:**
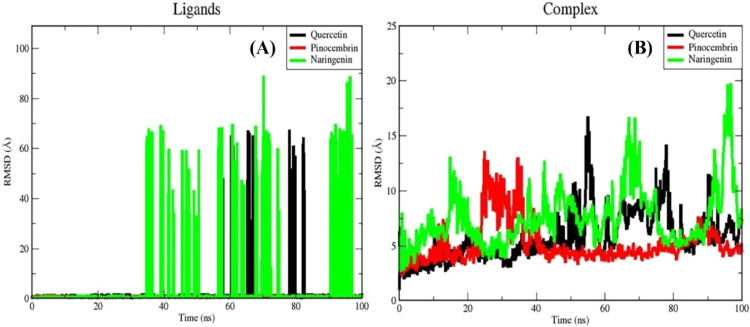
(A) Root Mean Square Deviation (RMSD) plot showing the structural stability of the ligands quercetin (black), pinocembrin (red), and naringenin (green) throughout the 100 ns simulation; (B) RMSD plot of the protein-ligand complexes highlighting the dynamic stability of quercetin (black), pinocembrin (red), and naringenin (green) within the ZIKV envelope protein binding site over time.

Fig. 9A shows the RMSD of the quercetin-ZIKV_E, pinocembrin-ZIKV_E, and naringenin-ZIKV_E complexes. Up to 35 ns, all ligands remained stably bound. After this point, quercetin and naringenin exhibited marked increases in RMSD, reflecting multiple binding orientations and occasional movement beyond the binding pocket. In contrast, pinocembrin maintained a stable orientation with minimal fluctuation (average RMSD: 1.101 Å).

However, after 35 ns, the naringenin and quercetin ligands showed a sharp increase in RMSD values. The naringenin ligand achieved the highest RMSD increases in the simulation between 65–70 ns and 90–95 ns. Similarly, the quercetin ligand exhibited the highest RMSD values, particularly at 65 ns and 80 ns during the simulation. These findings indicate that both naringenin and quercetin, when bound, exhibit numerous binding orientations and move beyond the binding pocket during the MD simulations. Both the quercetin and naringenin ligands changed the direction of their binding during the simulation. The pinocembrin ligand, on the other hand, stayed in its binding state the whole time. This observation indicates that the intermolecular interactions of the pinocembrin compound remain unchanged during the simulation, and its conformational orientation remains consistent without significant fluctuation.

We calculated the RMSD values of all three protein-ligand complexes throughout the 100 ns MD trajectory to assess the stability of each complex, as shown in Figure 19B The average absolute RMSD values for the complexes are 15.65, 6.201, and 16.575 Å, respectively. All three complexes continued to move away from their initial positions until 20 ns after the start of the simulation, with this increase being particularly noticeable in the naringenin complex. After this period, there was a notable decrease in the naringenin complex. While it remained stable for a short time, the RMSD value began to gradually increase after 35 nanoseconds (ns), reaching its peak values, particularly between 60–80 ns and the last 10 ns of the simulation. The quercetin complex showed a similar behavior throughout the simulation. The RMSD value showed the highest increase, especially after 40 ns, and continued to fluctuate until the end of the simulation. However, the pinocembrin complex showed a different stability compared to the other two structures. Although the pinocembrin complex showed a sharp increase at 20 ns, it remained stable from this point until the simulation's end. Overall, the pinocembrin complex with the ZIKV envelope protein (PDB ID 5JHM) demonstrated the lowest RMSD value compared to the complexes with quercetin and naringenin complexes. This indicates that the pinocembrin complex is more stable and undergoes fewer conformational changes than the other complexes.

#### 3.7.2 Root mean square fluctuation (RMSF) analysis

**Fig. 10:**
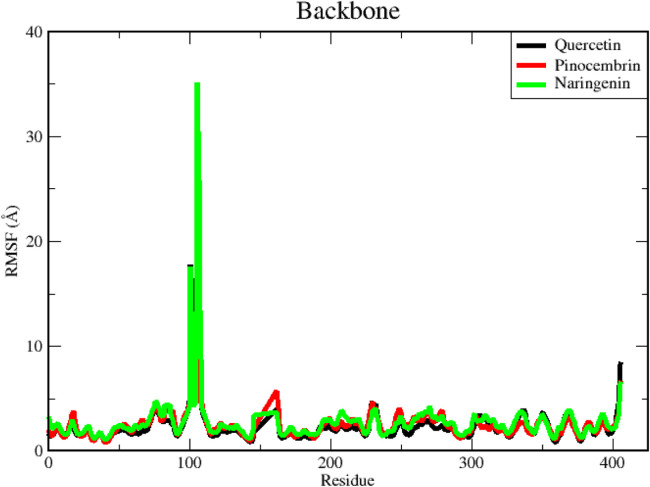
Residue-level RMSF analysis of protein backbone in ligand-bound complexes. Per-residue fluctuation patterns showing flexible and stable regions of the protein backbone after ligand binding.

RMSF is a valuable method for assessing the residual flexibility of a protein's backbone, which is an important aspect of MD modeling. The RMSF metric evaluates the crucial role of protein residues in achieving a stable conformation in a protein-ligand complex. A higher RMSF value indicates greater flexibility, while lower RMSF values indicate a more stable region (Karjiban et al., 2009). Conversely, smaller RMSF values at binding-site residues indicate reduced mobility consistent with stable ligand engagement; they should not be interpreted as a loss of interaction capacity. In contrast, larger RMSF values denote higher flexibility and local instability (Shukla and Tripathi, 2020). In this study, we generated RMSF values to evaluate how ligands affect the residual flexibility of the protein backbone, as shown in Fig. 10.

The RMSF plot produces a graphical representation characterized by distinct peaks, with each peak corresponding to a relatively high RMSF value (Fig. 10). According to the RMSF plot, the compound pinocembrin has the lowest RMSF value for the protein backbone, whereas the compounds quercetin and naringenin have larger RMSF values. We detected a single point with a maximum peak in all three complexes, situated within the range of amino acids. Interestingly, studies have shown that these amino acids do not form part of the binding pocket and do not significantly influence the binding process. However, the peak in the naringenin complex is significantly higher compared to the other two complexes. Here it is clear that the ligand-protein interactions in the naringenin complex have a less stable binding, as indicated by the higher RMSF values. Fig. 10 also shows that the RMSF value at the N-terminal residues (ends) is quite high for each complex. This is because these residues are the protein structure's highly reactive and free-to-move tails or ends. Each of the three residues of the complex system, especially THR406 (N-terminal), displays significantly larger changes compared to the other residues. This is likely due to its positioning at the terminus of the protein sequence, which grants it greater flexibility compared to other amino acid residues. As depicted in Fig. 10, the RMSF value of the N-terminal end of quercetin and naringenin compounds is significantly higher than that of the pinocembrin compound.

#### 3.7.3 Radius of gyration (Rg) analysis

We plotted the gyration radius diagram of each structure throughout the simulation to evaluate the changes in structural compression. We calculated Rg to ascertain the system's compactness over time, where a higher Rg value signifies less compactness (more unfolded) with conformational entropy, and a lower Rg value indicates more compactness with increased structural stability (more folded) (Chen et al., 2020).

**Fig. 11:**
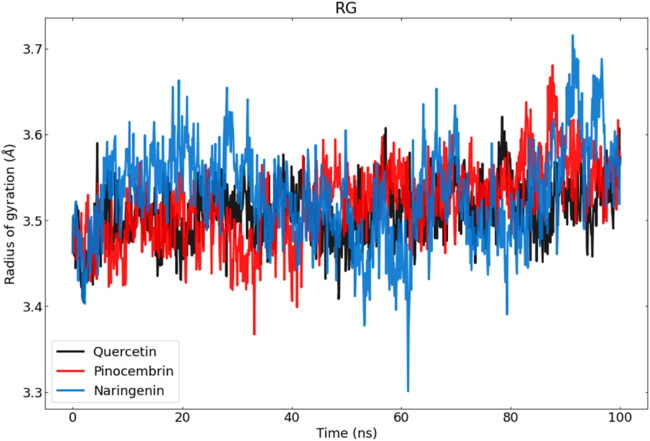
Radius of Gyration (Rg) analysis of ZIKA vius protein–ligand complexes throughout the simulation. Evaluates structural compactness and conformational integrity of protein–ligand systems over 100 ns.

In general, the fluctuations observed in the Rg values exhibit different characteristics for each complex, and their structural compactness is quite different. According to the results, complex-naringenin exhibits the largest Rg value and suffers a greater loss of compactness compared to the other complexes. During the first 40 nanoseconds and after the simulation, the RG value increased significantly, indicating a relatively low binding affinity between the ligand and the active site. In contrast, the pinocembrin complex and the naringenin complexes exhibit lower RG values. The pinocembrin complex exhibited considerable fluctuations during the simulation, particularly between 80 and 90 nanoseconds. However, it ultimately maintained its stability and compactness until the end of the simulation (Fig. 11).

#### 3.7.4 Hydrogen bond analysis

**Fig. 12:**
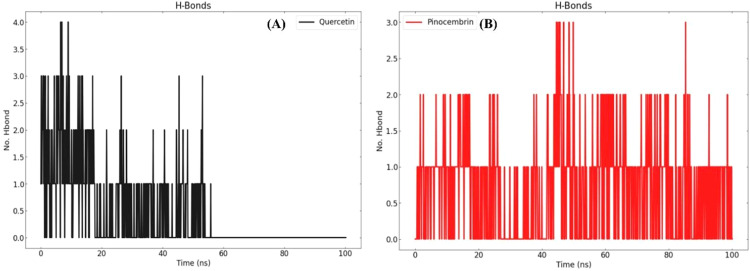
Hydrogen bond formation timeline during molecular dynamics simulations for (A) quercetin and (B) pinocembrin.

We quantified hydrogen bonds (H-bonds) occupancy/time evolution, then we interpreted those data together with RMSD/RMSF/Rg to understand stability during the simulation. To comprehend the relationships between biomolecules, it is necessary to perform a geometric study of hydrogen bonds. Hydrogen bonds play a crucial role in preserving the structural integrity of biomolecules. During MD simulations, the formation of hydrogen bonds (H-bonds) is crucial for the stability of the complexes. We calculated and plotted the number of H-bonds formed by the ligand molecule with the proteins (quercetin and pinocembrin) on a graph, as shown in Fig. 12 **(A and B)**.

We used the VMD H-bonding analysis program to investigate all possible H-bonding interactions between the protein and the ligand over a period of time. The result calculates the cumulative count of hydrogen bonds and their presence for a given time period. The numbers can be obtained from the "Percentage occupancy of the Hbond" output of the H-bond analysis tool. The output of the H-bond analysis tool provides the numbers for the percentage occupancy of the H-bond.

The MD simulations preserved not all hydrogen bonds present in the docking structures, but they also observed the formation of new hydrogen bonds. The MD simulation of the naringenin complex detected hydrogen bonds. Conversely, the quercetin molecule retained the majority of hydrogen bonds observed in the docking simulation, whereas the pinocembrin compound had a lower number of such bonds (Table 7).

**Table 7:** Hydrogen bonding interactions and occupancy percentage in protein–ligand complexes. List of key donors–acceptor pairs and the percentage of time these hydrogen bonds persisted during MD simulation.

Compound	Donor	Acceptor	Occupancy
Quercetin	MET345-Main-N	UNK0-Side-O6	3.29 %
	UNK0-Side-O6	ASP348-Main-O	3.19 %
Pinocembrin	UNK0-Side-O3	HSP144-Main-O	33.13 %
	UNK0-Side-O3	ALA361-Main-O	10.08 %

During the simulation, the quercetin molecule had a lower number of hydrogen bonds, with the majority occurring between 10 ns and 30–58 ns. However, the pinocembrin molecule exhibited a higher degree of hydrogen bond formation, and this formation persists throughout the entire simulation. These hydrogen bonds reached their highest value between 40–60 ns and 80–90 ns, especially in the simulation. The quercetin complex remained stable throughout the molecular dynamics (MD) due to its connections with the amino acids MET345 and ASP348, whereas the pinocembrin complex maintained its connections with HSP144 and ALA361. As a result, the pinocembrin compound has a lower total number of hydrogen bonds but a higher hydrogen bond occupancy than the quercetin compound. Because the number and occupancy of hydrogen bonds are key for stabilizing the interaction of the protein-ligand complex, the pinocembrin complex is a highly stable system.

#### 3.7.5 Principal component and free energy landscape analysis

**Fig. 13:**
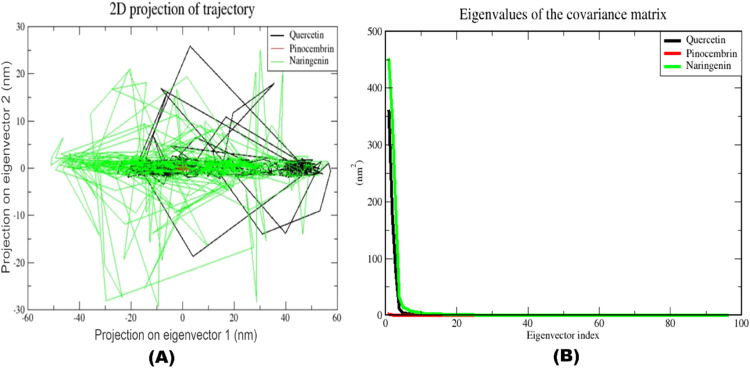
(A) 2d projections of trajectories on eigenvectors of quercetin, pinocembrin, and naringenin. (B) Cumulative variance explained by PC1 and PC2 for quercetin, pinocembrin, and naringenin. Quercetin (black), Pinocembrin (red), and Naringenin (green) complexes.

We used Principal Component Analysis (PCA) to analyze the simulation trajectory and identify the main modes of motion and generated PCA graphs to analyze the primary structural variations of the quercetin, pinocembrin, and naringenin complexes. Fig. 13 **(A and B**) displays the collective motion of all the protein-ligand complexes (PLCs) through the first two principal components (PCs) and their 2D projections of PC1 and PC2. The first two eigenvectors were projected onto each other by alphacarbon. Compared to the quercetin and naringenin complexes, the two main components of the pinocembrin complex (PC1 and PC2) showed that this complex covers a smaller region in the conformational space, whereas the naringenin complex covers the largest conformational space, indicating an overall increase in the flexibility of the protein after ligand binding, but after binding to pinocembrin, the target protein stabilized and occupied a small region in the conformational space, indicating a loss of flexible movements of the protein (Fig. 13).

In the figure of Fig. 13b, labels on each point indicate the cumulative total of variance attributed to a certain eigenvector and its preceding eigenvectors. Cumulative variance is depicted as a function of the number of principal components (PCs). As can be seen from Fig. 13, initially the intrinsic values of quercetin and naringenin complexes were associated with larger coordinated movements, while no such change was observed in the pinocembrin complex.

#### 3.7.6 Gibbs free energy landscape (FEL)

We used Gibb's free energy landscape (FEL) to better understand the general conformational behavior of the system. To identify the low-energy basins (minima) investigated during the simulation, we conducted an FEL analysis (Abouzied et al., 2024). The FEL was plotted in both 2D and 3D using PC1 and PC2. **Figure S1B** displays the free energy landscape for a duration of 100 nanoseconds. The color bar shows the Gibbs free energies (kcal/mol) of the different structural states, with blue being the lowest energy state and dark yellow the highest energy state. The simulation demonstrated that the protein-ligand combination is not very stable, as evidenced by the shallow and narrow energy basin.

The stability of the corresponding conformation and system improves as the Gibb's free energy value decreases. The findings indicate that the pinocembrin complex has a larger number of conformations in a low energy state compared to naringenin and quercetin. The dark blue patches in **Figure S1** represent these conformations. Furthermore, the pinocembrin complex exhibited two distinct and extensive valleys, whereas the other two compounds showed a less pronounced and narrower energy basin. This indicates that the pinocembrin complex possesses exceptional stability (**Figure S1 A-C**).

#### 3.7.7 Dynamics cross-correlation matrices (DCCM) analysis

We used DCCM analysis to investigate the conformational changes of the targeted ZIKV envelope, analyzing all *Ca* atoms. The 2D plots of the DCCM revealed a strong correlation in the movements of the residues throughout the simulation procedure, as depicted in **Figure S2**. The DCCM exhibited a comprehensive correlation ranging from −1.0 to 1.0, with dark blue representing the lowest correlation and dark red the highest correlation. We used different shades of color to distinguish the various levels of correlation between the residues, with the intensity of the color directly corresponding to the strength of the association. A positive correlation (from 0 to 1) indicates that the residues move in the same direction, whereas a negative correlation (from −1 to 0) indicates that the residues are moving in the opposite direction.

When comparing the DCCM diagrams of the three systems, it becomes evident that the correlation behavior of the numbered systems naringenin and quercetin differs drastically from that of the pinocembrin system. The black boxes reveal a notable rise in negative correlation movements in both systems, while the positive correlation decreases in comparison to the pinocembrin system. This rise is particularly noticeable in the structure of naringenin (**Figure S2 A-C**).

This increase indicates significant changes in protein-related motions following ligand binding. Analysis of the DCCM diagrams for the quercetin and naringenin systems revealed no significant differences in the correlation motions. The fact that the naringenin and quercetin systems have negatively correlated motions shows that the protein's structure is very unstable, especially after it comes into contact with a ligand.

These results link the DCCM results to the biological functionality of the ZIKV envelope protein. The increase in negative correlations observed for the naringenin and quercetin systems, particularly in regions critical for conformational rearrangements, suggests that these ligands may destabilize the dynamic flexibility required for the protein’s functional transitions. This destabilization likely impairs the ability of the envelope protein to undergo the structural rearrangements required for membrane fusion and viral entry. In contrast, the relatively stable correlation patterns in the pinocembrin system suggest that this ligand maintains the conformational integrity of the protein to a greater extent, which may correspond to a less pronounced inhibitory effect. These observations highlight the influence of ligand binding on the functional dynamics of the ZIKV coat protein, with quercetin and naringenin showing a greater potential to disrupt its biological activity.

#### 3.7.8 Binding free energy analysis

We used the MM/PBSA approach to calculate the binding free energy in protein-ligand complexes (quercetin-ZIKV_E, pinocembrin-ZIKV_E, and naringenin-ZIKV_E) for the analysis of molecular binding relationships. This approach takes into account several interactions, such as binding and non-binding forces, like van der Waals and electrostatic forces. The binding free energy of these complexes was calculated using the MM/PBSA approach, focusing on the last 10 nanoseconds of the trajectory. We employed the MM/PBSA approach to determine the binding free energy (DG bind) of the ligands quercetin, pinocembrin, and naringenin. MM/PBSA values for the less stable complexes should be interpreted comparatively, as they reflect equilibrated sub-windows rather than the entire trajectory. Although a larger amount of frames could be selected for this step, it would come at the risk of including highly unstable conformations from our 100 ns MD, so preference was given to the last, and theoretically more stable, frames; moreover, as this analysis is purely comparative, as long as the same amount of frames is selected for each system, the overall behavior should remain similar compared to more lasting calculations. In this approach the binding affinity values are integrated. Lower DG values suggest higher binding affinities between proteins and ligands.

As the values become increasingly negative, the free energy between proteins and ligands becomes more favorable. Table 8 and **Figure S3 (A-C)** present the free energies of the ligands. The results show that the quercetin complex has a free energy of −0.03 ± 4.88 kJ/mol, the pinocembrin complex has a free binding energy of −8.27 ± 2.50 kJ/mol, and the naringenin complex has a free binding energy of −0.13 ± 4.14 kJ/mol. This indicates that the pinocembrin complex has the highest binding free energy when compared to the quercetin and naringenin complexes.

**Table 8:** Binding free energy decomposition of flavonoid-ZIKV_E complexes based on mm-pbsa calculations. Displays the contribution of van der Waals (ΔE_VDW_), electrostatic (ΔE_EEL_), polar solvation (ΔG_PB_), non-polar solvation (ΔG_NP_), and dispersion energy (ΔG_DISP_) to the total binding free energy (ΔG_Binding_) for each ligand, calculated via the MM-PBSA approach.

Name	ΔE_VDW_ (kJ/mol)	ΔE_EEL_ (kJ/mol)	ΔG_PB_ (kJ/mol)	ΔG_NP_ (kJ/mol)	ΔG_DISP_ (kJ/mol)	ΔG_Binding_ (kJ/mol)
Quercetin	−0.00	−0.01	−0.02	0.00	0.00	−0.03±4.88
Pinocembrin	−30.85	−11.44	37.27	−3.25	0.00	−8.27±2.50
Naringenin	−0.00	0.00	−0.13	−0.00	0.00	−0.13±4.14

#### 3.7.9 Normal mode analysis

We investigated the dynamic behavior of the ZIKV envelope protein in complex with naringenin, pinocembrin and quercetin using the IMODS platform. The analysis showed how each of these compounds affects the flexibility and stability of the receptor, which may be consistent with the other analyzes developed in this work. These results act as a complement to our MD findings, allowing us to explore longer simulated timeframes at low computational costs when compared to more robust dynamic simulations. These results are used to predict how a given system would behave over longer periods of time, expanding further than regular dynamics.

The eigenvalue analysis of the three complexes reveals distinct differences in the flexibility of the ZIKV envelope protein upon ligand binding. Quercetin stands out as it induces the most flexible state of the protein, as evidenced by the lowest eigenvalue (5.803972 × 10^−05^) **(Figure S4 A).** This suggests that the binding of quercetin allows the protein to undergo more significant conformational changes with minimal energy investment, a property that could be crucial for its function as a therapeutic agent. In contrast, pinocembrin with a higher intrinsic value (8.319865 × 10^−05^) **(Figure S5A)** appears to confer a more rigid conformation to the receptor, suggesting that more energy would be required to effect structural changes. Naringenin shows an intermediate effect in promoting flexibility, but not to the extent observed with quercetin (6.945799 × 10^−05^) **(Figure S6A)**.

While our findings suggest that quercetin induces the highest degree of flexibility in the ZIKV envelope protein, as evidenced by the lowest eigenvalue (5.803972 *x* ^−05^), it's essential to consider contrasting studies that offer a different perspective on the effects of flavonoids on protein dynamics. A study on the structure-activity relationship between quercetin and naringenin has shown that Quercetin's greater number of hydroxyl groups and its structural planarity due to a double bond in ring A contribute to its distinct binding mode and interaction forces, which might not always translate to increased flexibility but rather to specific, stronger interactions at the binding site​ (Tu et al., 2015).

The variance distribution further corroborates our observations. While all three compounds allow the receptor to explore its conformational landscape, Quercetin facilitates a broader range of motion, with nearly 90 % **(Figure S4B)** of the cumulative variance captured within the first nine modes. This suggests that Quercetin binding imparts a higher degree of conformational freedom, which could be beneficial for adapting to various functional states. Naringenin and Pinocembrin, with cumulative variances around 80 % **(Figure S5B)**, still permit substantial flexibility, though they likely stabilize specific conformations to a greater extent than Quercetin **(Figure S6B)**, However, contrasting studies show that a greater range of motion does not always equate to better therapeutic efficacy. In some cases, too much flexibility can lead to an increase in non-specific interactions, which can reduce the selectivity of the drug and increase the likelihood of off-target effects (Donyapour et al., 2023).

The covariance matrices provided a deeper understanding of the dynamics between residues within the ZIKV envelope protein when complexed with these flavonoids. Quercetin again showed a pronounced effect, triggering extensive correlated and anticorrelated movements between residues **(Figure S4C, S5C)**. These dynamic couplings suggest that the binding of quercetin can significantly alter the internal communication pathways within the protein, potentially affecting how the receptor interacts with other molecules or exerts its biological functions. Naringenin and Pinocembrin **(Figure S6C)** also influence the protein's dynamic network, but with less dramatic shifts compared to Quercetin.

The elastic network models for these complexes show important differences in the structural rigidity conferred by each flavonoid **(Figure S4D, S5D and S6D)**. Quercetin, which promotes the most flexible receptor conformation, is associated with a sparse network of stiff plumes, particularly in regions critical for maintaining structural integrity, suggesting that quercetin may enable the receptor to switch more easily between different functional states (Kerna et al., 2024). On the other hand, pinocembrin and naringenin appear to maintain more regions of structural stiffness, with denser networks of stiff springs observed in their respective models. This balance between flexibility and rigidity is crucial, as excessive flexibility could lead to a loss of functional specificity, while excessive rigidity could hinder the necessary conformational changes required for effective receptor function (Donyapour et al., 2023)​.

#### 3.7.10 QM/MM results

The energetic profiles of the three principal flavonoid–ZIKV_E complexes - quercetin–ZIKV_E, pinocembrin–ZIKV_E, and naringenin–ZIKV_E - were comprehensively evaluated using QM/MM calculations. The results revealed a marked enhancement in system stability following QM/MM optimization, most notably for the quercetin–ZIKV_E complex. Prior to the QM/MM refinement, quercetin–ZIKV_E already exhibited the lowest quantum mechanical energy (−13,978.515 Ha), suggesting inherent structural stability relative to the other complexes. Post-optimization, the energy further decreased substantially to −71,718.787 Ha, indicating a pronounced stabilization effect mediated by the QM/MM interaction (Table 9)**.**

**Table 9:** QM/MM optimization and energy minimization data for flavonoid–zikv envelope protein complexes. Initial and final quantum mechanical (QM) energies (in Hartrees, Ha) and QM/MM interaction energies (in kcal/mol) for naringenin, pinocembrin, and quercetin complexes with the ZIKV envelope receptor, obtained through ONIOM-based hybrid quantum mechanics/molecular mechanics (QM/MM) calculations.

	Naringenin	Pinocembrin	Quercetin
Initial (*Final*) Quantum Energy in Ha	−13,890.064 (−29,575.465)	−13,849.426 (−29,580.622)	−13,978.515 (−71,718.787)
Initial (*Final*) QM/MM Interaction Energy in Kcal/mol	105.98 (−69.146)	104.04 (−56.425)	−27.244 (−67.917)

This energetic profile is consistent with previous findings that underscore quercetin’s high binding affinity and its pronounced capacity to stabilize protein conformations through an extensive network of hydrogen bonds and hydrophobic contacts (Zhang et al., 2024; Shaw et al., 2025). The structural adaptability and interaction versatility of quercetin underpin its broad-spectrum biological activities, encompassing antiviral, anticancer, and anti-inflammatory properties. Notably, the highly negative QM/MM interaction energy observed for the quercetin–ZIKV_E complex reflects a thermodynamically favorable and robust binding event, thereby reinforcing its potential as a promising therapeutic candidate (Shaw et al., 2025).

In comparison, both naringenin–ZIKV_E and pinocembrin–ZIKV_E demonstrated enhanced energetic stability following QM/MM optimization, with their Quantum energies decreasing from −13,890.064 to −29,575.465 and from −13,849.426 to −29,580.622, respectively. Nonetheless, the substantially lower final energy observed for the quercetin–ZIKV_E complex underscores its superior stability, likely reflecting more extensive and effective binding interactions - an observation aligned with previous molecular docking studies. These findings are consistent with literature reports describing the moderate binding affinities of naringenin and pinocembrin and their therapeutic relevance in contexts such as neuroprotection and cardiovascular modulation (García Forero et al., 2019; Habtemariam, 2019; Borriello et al., 2019).

A critical insight emerges when comparing the initial and post-optimization QM/MM interaction energies across the three flavonoid–ZIKV_E complexes. All compounds exhibited a marked decrease in interaction energy following optimization, indicating enhanced binding stability - a desirable and significant outcome. Initially, both naringenin–ZIKV_E and pinocembrin–ZIKV_E presented positive interaction energies (105.98 and 104.04 kcal/mol, respectively), denoting thermodynamically unfavorable binding when evaluated in their pre-optimization, force field-based docking conformations.

This observation underscores a pivotal point: despite favorable docking scores, the most promising docked complexes can be intrinsically unstable when examined through a quantum mechanical lens. The electronic structure analysis revealed inconsistencies between apparent docking affinity and true energetic viability, exposing limitations inherent in classical force field-based docking approaches. Therefore, we emphasize the critical importance of performing quantum-level optimization following preliminary molecular docking procedures to obtain more accurate and energetically reliable representations of biomolecular interactions.

After optimization, all complexes reached negative interaction energies: −69.146 kcal/mol for naringenin, −56.425 kcal/mol for pinocembrin, and −67.917 kcal/mol for quercetin. The transition from positive to negative interaction energy for naringenin and pinocembrin confirms that QM/MM refinement substantially improved binding stability (Table 9)**.**

#### 3.7.11 Interaction analysis of flavonoid–ZIKV_E complexes after QMMM calculations

**Figures S7A, S7B** and **S7C** describe the target site to which the drug molecules bind during the formation of the complex structures and molecular docking. Structural analysis of the interactions between the flavonoids and the target protein interferes with the key residues in the active site of the protein and forms specific hydrogen bonds that stabilize the complex.

In the case of naringenin **(Figure S7A)**, the molecule forms hydrogen bonds with ASN362, VAL303 and HIS144. These interactions are crucial for the stabilization of naringenin within the binding pocket. The moderate binding affinity observed here suggests that although naringenin can effectively bind to the protein, its influence on protein dynamics may be less pronounced compared to other flavonoids. This is consistent with the intermediate effects observed in previous analyzes, suggesting that naringenin may offer a balanced therapeutic potential.

Pinocembrin **(Figure S7B)** exhibits a more complicated interaction network, forming hydrogen bonds with LEU145, ILE365 and LYS301. The presence of multiple stabilizing interactions suggests a stronger and more specific binding affinity, which could lead to a stronger modulation of the protein's function. The ability of pinocembrin to bind multiple residues simultaneously could increase its efficacy in therapeutic applications, especially when stabilization of specific protein conformations is desired.

Quercetin **(Figure S7C)**, on the other hand, exhibits the most extensive interaction network, with hydrogen bonds forming between ASN362, VAL355 and LYS38. This robust binding suggests that quercetin has a higher affinity for the target protein, potentially leading to stronger biological effects. The strong and stable binding observed here supports the hypothesis that quercetin may be the most effective of the three flavonoids in modulating protein function, as it is consistent with previous results highlighting the significant impact of quercetin on protein flexibility and stability.

The binding interactions of naringenin, pinocembrin and quercetin with the target protein emphasize their potential as therapeutic agents, particularly in modulating protein functions relevant to diseases such as cancer and viral infections. The strong binding affinity of quercetin, for example, supported by multiple hydrogen bonds, is consistent with its known antioxidant and anti-inflammatory properties, which have been shown to inhibit critical enzymes involved in viral replication and carcinogenesis (EFSA Panel on Food Additives and Flavourings (FAF) 2024). Similarly, although pinocembrin and naringenin have different interaction profiles, they show promising therapeutic potential due to their unique binding mechanisms, which could be exploited for targeted therapy in various diseases, including neuroprotection and cardiovascular health (EFSA Panel on Food Additives and Flavourings (FAF) 2024).

### 3.8 Selectivity of flavonoids (Quercetin, pinocembrin, naringenin) for viral *versus* human proteins

Evaluating the selectivity of antiviral compounds for viral proteins *versus* human proteins is critical for understanding their therapeutic potential and minimizing off-target effects. However, literature specifically addressing the selectivity of flavonoids such as quercetin, pinocembrin, and naringenin for viral *versus* human proteins remains limited. Most existing studies focus primarily on the antiviral potency of flavonoids without comprehensive evaluation of their potential interactions with human protein targets.

One pertinent study, although focusing on a different flavonoid (silibinin), provides insights into how selectivity assessments can highlight the therapeutic promise of flavonoids. Silibinin exhibited strong selectivity for SARS-CoV-2 viral proteins such as the spike protein (IC50 0.029 μM), main protease (IC50 0.021 μM), and RNA-dependent RNA polymerase (IC50 0.042 μM), without any inhibitory effect on the human transmembrane serine protease TMPRSS2. Notably, silibinin was safe for mammalian cells at concentrations significantly higher than its antiviral IC50, underscoring its high therapeutic index (Hamdy et al., 2022). Although silibinin differs chemically from quercetin, pinocembrin, and naringenin, these findings illustrate the importance of selectivity analyses in flavonoid-based antiviral research.

Additionally, studies focusing on flavonoids such as quercetin have predominantly investigated their binding properties to specific human proteins, such as human serum albumin (HSA), rather than evaluating their preferential selectivity toward viral proteins. Quercetin’s binding affinity to HSA sites has been shown to be influenced significantly by structural modifications, but without a direct comparison against viral proteins, the clinical relevance for antiviral specificity remains uncertain (Mukai et al., 2024). Consequently, a direct extrapolation of such human-protein binding studies to antiviral selectivity must be approached cautiously.

The broader flavonoid literature demonstrates potential antiviral activities but generally lacks explicit comparative selectivity evaluations against human proteins. For instance, virtual molecular docking models have demonstrated quercetin's effective interaction with SARS-CoV-2 replication-cycle proteins, including spike, 3CLpro/MPro, and RdRP, highlighting multiple potential antiviral targets (Toigo et al., 2023). Additionally, quercetin derivatives have shown therapeutic potential against the Nipah virus target protein (Berkane et al., 2024), suppressing Porcine Epidemic Diarrhea Virus infection through inhibition of 3C-like protease activity at non-cytotoxic concentrations (Li et al., 2020), and significantly reducing Junín virus multiplication by affecting early viral adsorption and internalization steps (Alvarez De Lauro et al., 2023).

Similarly, naringenin has been reported to prevent ZIKV infection in human A549 cells effectively in a concentration-dependent and ZIKV-lineage-independent manner, with molecular docking suggesting potential interactions with the protease domain of the NS2B-NS3 protein (Cataneo et al., 2019). Prenylated derivatives of naringenin have also demonstrated potent anti-influenza virus activity, where cellular uptake varied significantly depending on the position of the prenyl group (Morimoto et al., 2022).

While our study provides an in-depth analysis of flavonoid interactions specifically with the ZIKV envelope protein, we did not directly assess their selectivity against human proteins or other off-target receptors. Although our *in silico* ADMET profiles indicated favorable pharmacokinetics and low predicted toxicity, these evaluations do not replace targeted selectivity analysis. Potential off-target interactions, which could lead to undesirable side effects or reduced antiviral efficacy, remain a critical consideration for the clinical translation of these compounds.

Future investigations should therefore aim to expand our computational methodologies to systematically include selectivity profiling. Performing comparative docking and molecular dynamics simulations against a panel of representative human proteins, and possibly other viral targets, would significantly enhance the reliability and clinical relevance of flavonoid-based antiviral research. Furthermore, extensive virtual screening approaches employing cross-docking analyses and *in silico* target-panel assessments could effectively identify off-target interactions early in the drug development process, refining flavonoid candidates for enhanced specificity and minimal adverse effects. Such selectivity-focused analyses will be essential for validating and optimizing flavonoids as clinically viable antiviral agents with maximal therapeutic efficacy and safety.

## 4 Conclusion

This computational study was conducted to identify new inhibitors for the ZIKV. We focused on naturally occurring compounds known as flavonoids and employed molecular docking to evaluate their antiviral potential. Through *in silico* studies, including drug-likeness, ADMET, and pharmacokinetic analysis, all flavonoids successfully passed the screening process for the prediction of their antiviral effectiveness. We performed molecular docking experiments against ZIKV envelope protein (PDB ID 5JHM), to further validate their potential. Surprisingly, most of the compounds had strong binding interactions and binding energies ranging from −6.5 to −8.3 kcal/mol against the ZIKV envelope protein. These findings indicate their potential to effectively suppress the ZIKV. The binding interactions observed were characterized by hydrophobic and hydrophilic contacts, which further enhanced the compounds' binding energy. Moreover, *in silico* pharmacokinetic predictions yielded encouraging results suggesting improved pharmacokinetic profiles, favorable solubility, and a lack of toxic effects for all the compounds studied. The majority of the compounds inhibited CYP450 1A2, with the exception of taxifolin, which acted as a substrate of CYP450 2D6. Furthermore, the compounds' total clearance rate varied from 0.06 ml/min/kg to 0.566 ml/min/kg. While this study provides valuable insights into molecular interactions and binding affinities, it is limited to computational approaches, which, though useful for preliminary screening, require experimental validation. Our molecular dynamics simulations were conducted over 100 ns, highlighting the need for experimental studies to confirm binding affinities, assess antiviral activity, and evaluate pharmacokinetics and toxicity profiles. Extended simulations and enhanced sampling techniques could further refine our understanding of the dynamic behavior of these complexes. Future steps include experimental assays to validate inhibitory effects and formulation studies to improve bioavailability, paving the way for effective antiviral therapies against ZIKV.

## Ethics approval and consent to participate

Not applicable since it’s a theoretical study.

## Consent for publication

Not applicable.

## Clinical trial number

Not applicable.

## Availability of data and materials

The datasets generated and/or analyzed during the current study are available in the manuscript. However, some data are taken from online sources such as protein databank and they are available in this link (https://www.rcsb.org/structure/5jhm).

## Funding

None.

## CRediT authorship contribution statement

**Jehad Zuhair Tayyeb:** Writing – review & editing, Writing – original draft, Software, Resources, Methodology, Data curation, Conceptualization. **Maria Karolaynne da Silva:** Writing – original draft, Methodology, Formal analysis, Data curation, Conceptualization. **Aamal A. Al-Mutairi:** Writing – original draft, Methodology, Formal analysis, Data curation, Conceptualization. **Hanan M. Alharbi:** Writing – original draft, Visualization, Resources, Project administration, Formal analysis, Data curation. **Alaa A. Khojah:** Writing – review & editing, Software, Resources, Methodology, Formal analysis, Data curation. **Imren Bayıl:** Validation, Software, Resources, Methodology, Investigation, Formal analysis, Data curation. **Abdullah Yahya Abdullah Alzahrani:** Writing – review & editing, Visualization, Project administration, Investigation, Formal analysis, Data curation. **Zsolt Tóth:** Writing – review & editing, Project administration, Investigation, Formal analysis, Data curation. **Jonas Ivan Nobre Oliveira:** Writing – review & editing, Validation, Supervision, Software, Resources, Data curation, Conceptualization. **Magdi E.A. Zaki:** Writing – review & editing, Validation, Supervision, Software, Resources, Data curation, Conceptualization.

## Declaration of competing interest

The authors declare no conflicts of interest

## Data Availability

Data will be made available on request.

## Appendix Supplementary materials

Image, application 1
