# Supplementary material for: Evaluation of Potential Inhibitors of Zika Virus Envelope Protein Through Molecular Docking and Molecular Dynamics Simulation
Source: Virus Res. 2025 Sep 11;361:199630. doi: 10.1016/j.virusres.2025.199630 (PMC12481941; doi:10.1016/j.virusres.2025.199630)
Supplement: Supplementary file 1 [file mmc1.docx]

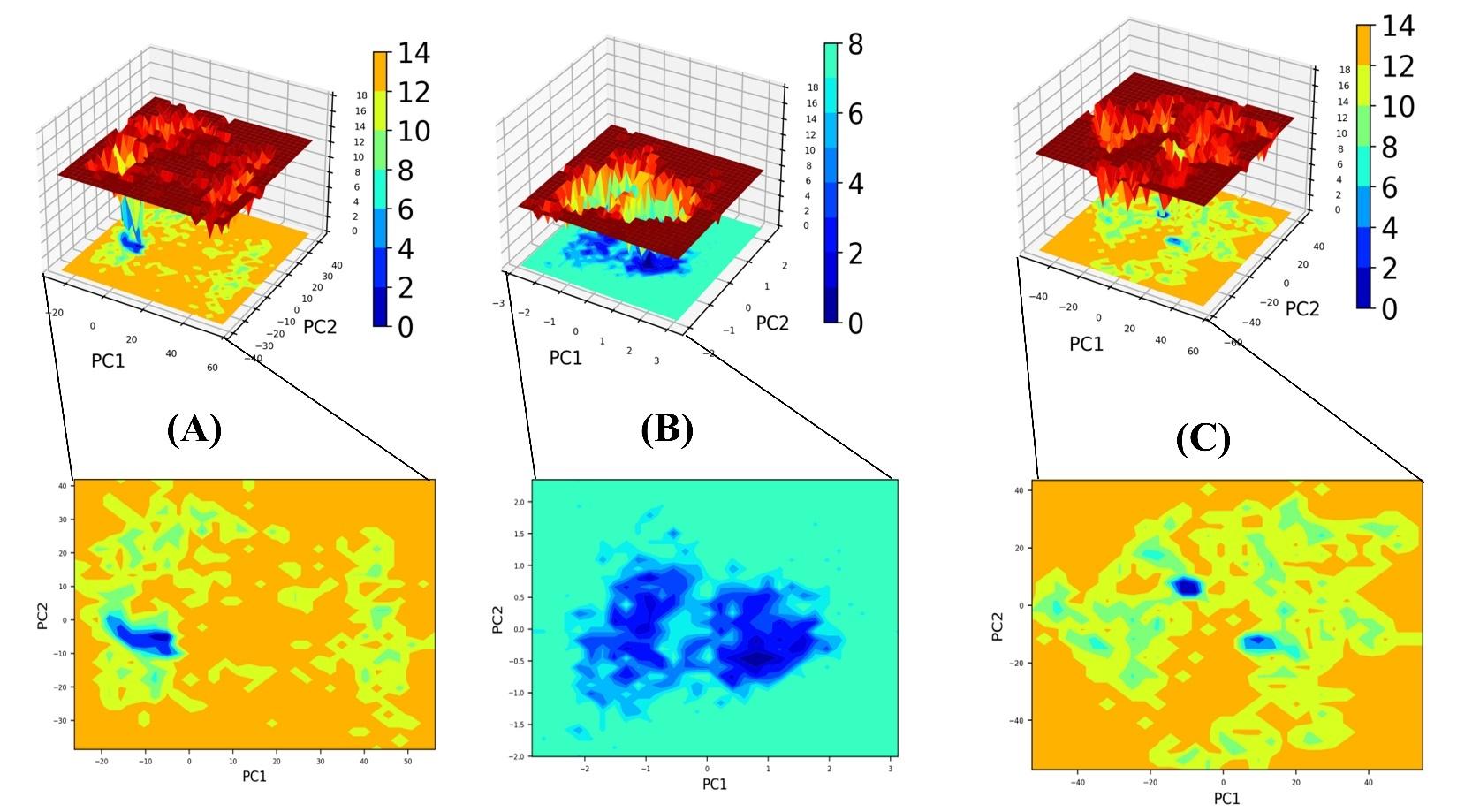


**Figure S1:** Analysis of the Gibbs free energy landscape of the principal components (PC1 and PC2) derived from the molecular dynamics simulations. The 3D energy surfaces and the corresponding 2D contour plots represent the conformational stability and the energy minimum of the ligand-protein complexes: (A) quercetin, (B) pinocembrin and (C) naringenin. The blue regions indicate low energy states corresponding to more stable conformations, while the yellow to red regions represent higher energy regions.


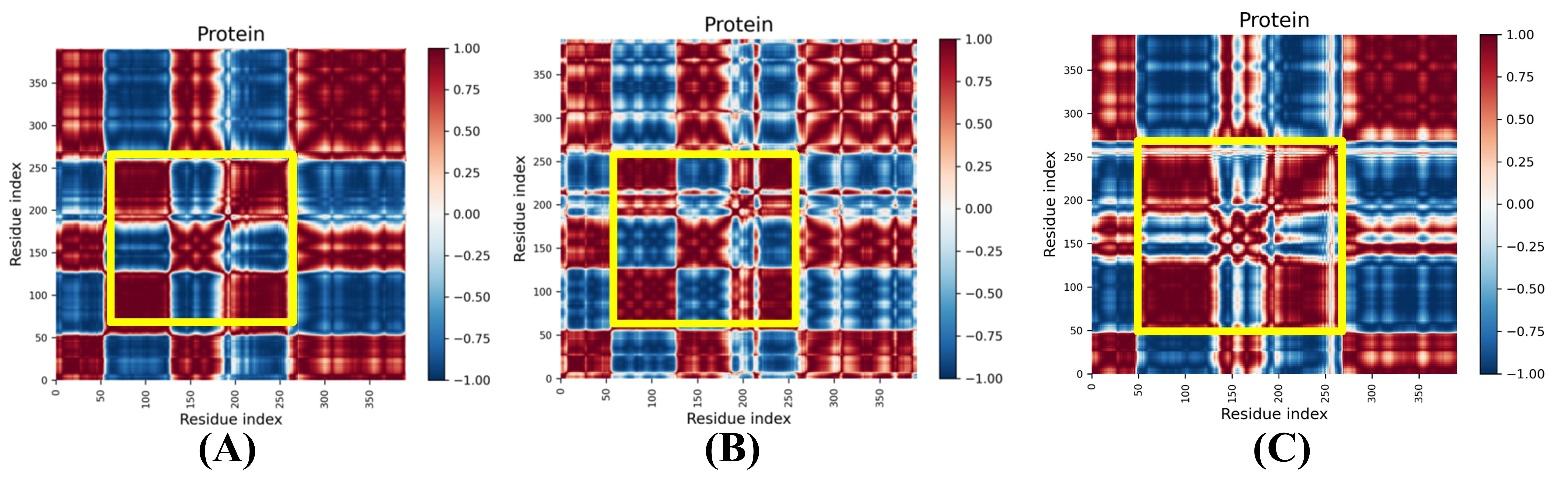


**Figure S2:** Dynamic cross-correlation matrix (DCCM) plots for (A) quercetin, (B) pinocembrin, and (C) naringenin.


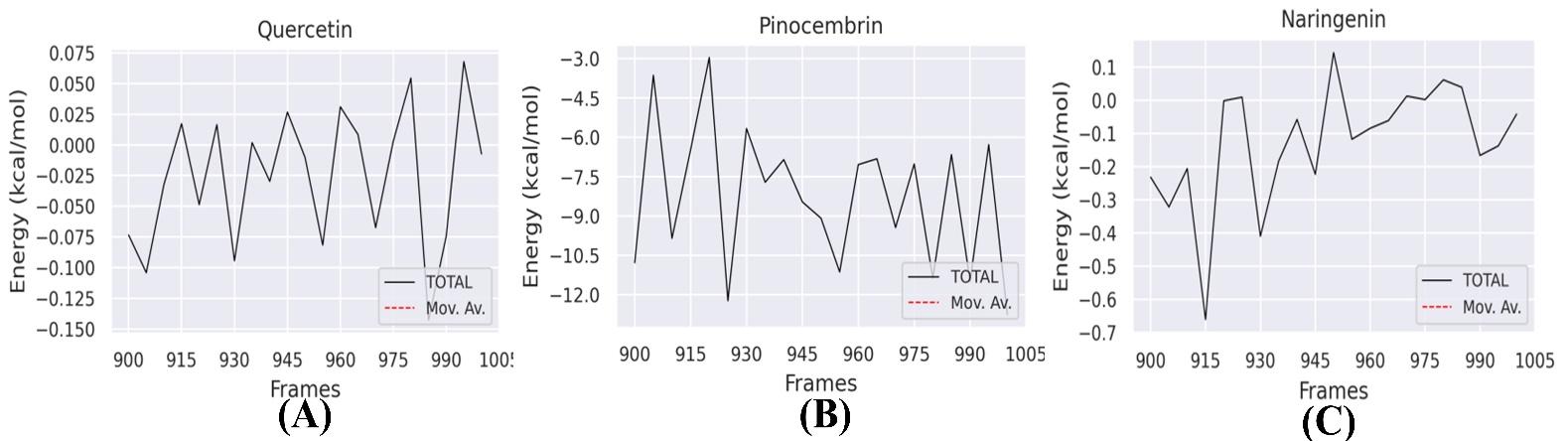


**Figure S3:** Binding free energy plots for (A) quercetin, (B) pinocembrin and (C) naringenin

**Table 8:** Binding free energy decomposition of flavonoid-ZIKV_E complexes based on mm-pbsa calculations. Displays the contribution of van der Waals (ΔE_VDW_), electrostatic (ΔE_EEL_), polar solvation (ΔG_PB_), non-polar solvation (ΔG_NP_), and dispersion energy (ΔG_DISP_) to the total binding free energy (ΔG_Binding_) for each ligand, calculated via the MM-PBSA approach/


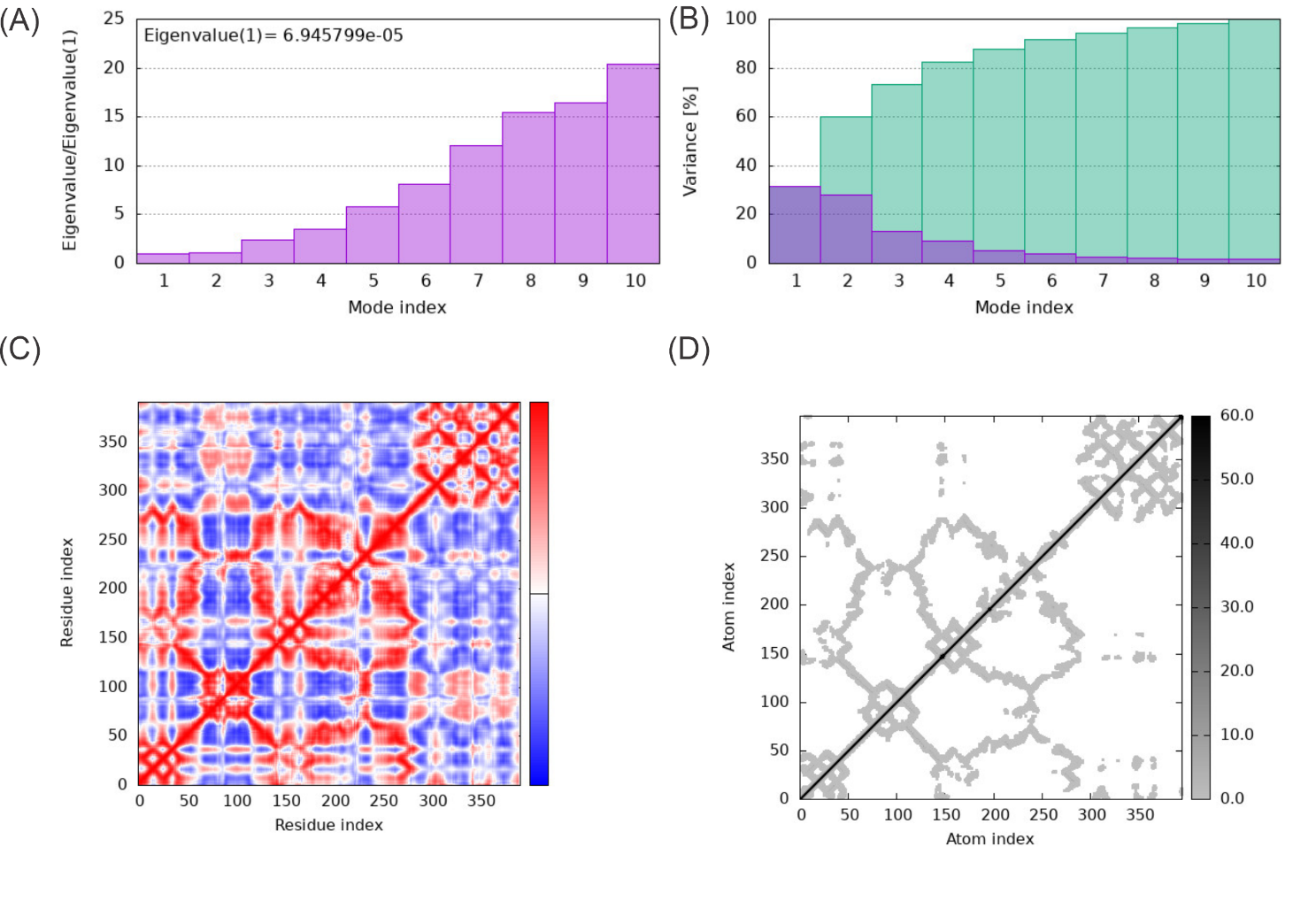


**Figure S4:** Normal mode analysis of the ZIKV envelope protein in complex with naringenin. (A) Eigenvalue spectrum indicating the relative magnitude of vibrational modes; (B) Cumulative variance explained by the top 10 modes; (C) Covariance matrix showing correlated (red) and anti-correlated (blue) residue motions; (D) Elastic network model highlighting atom–atom interactions and network rigidity across the protein structure.


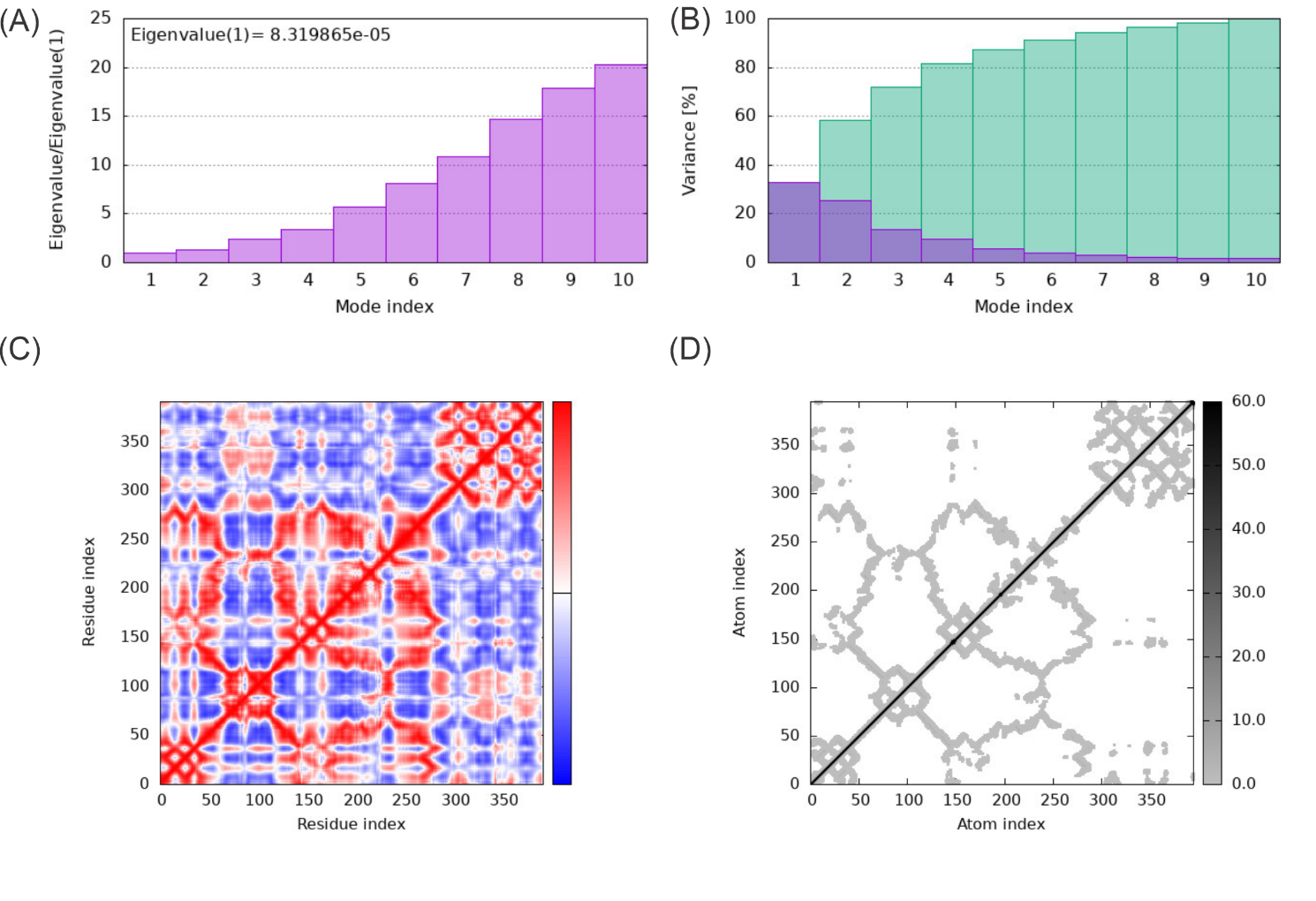


**Figure S5:** Normal mode analysis of the ZIKV envelope protein in complex with pinocembrin. (A) Eigenvalue spectrum indicating the relative magnitude of vibrational modes; (B) Cumulative variance explained by the top 10 modes; (C) Covariance matrix showing correlated (red) and anti-correlated (blue) residue motions; (D) Elastic network model highlighting atom–atom interactions and network rigidity across the protein structure.


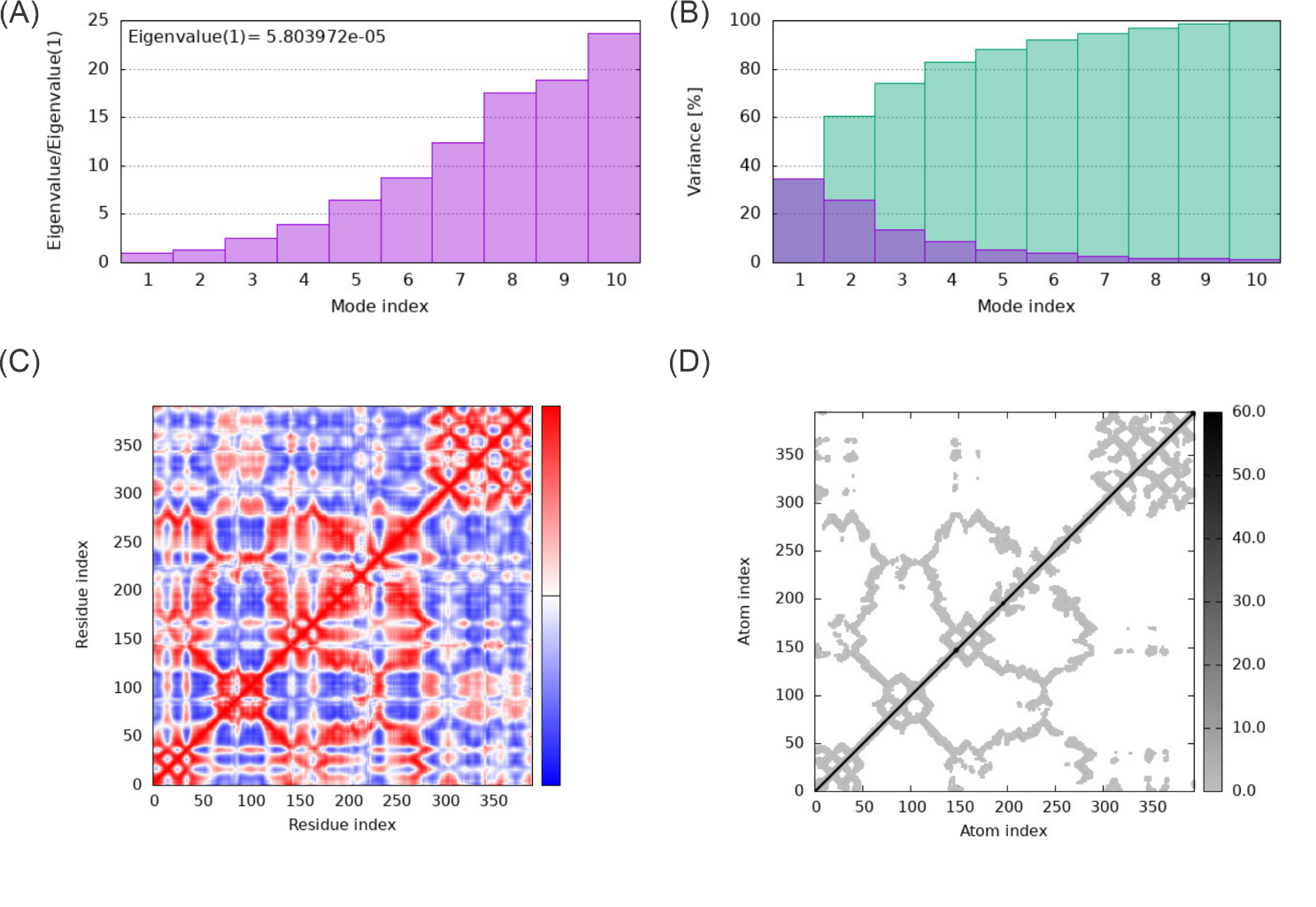


**Figure S6:** Normal mode analysis of the ZIKV envelope protein in complex with quercetin. (A) Eigenvalue spectrum indicating the relative magnitude of vibrational modes; (B) Cumulative variance explained by the top 10 modes; (C) Covariance matrix showing correlated (red) and anti-correlated (blue) residue motions; (D) Elastic network model highlighting atom–atom interactions and network rigidity across the protein structure.


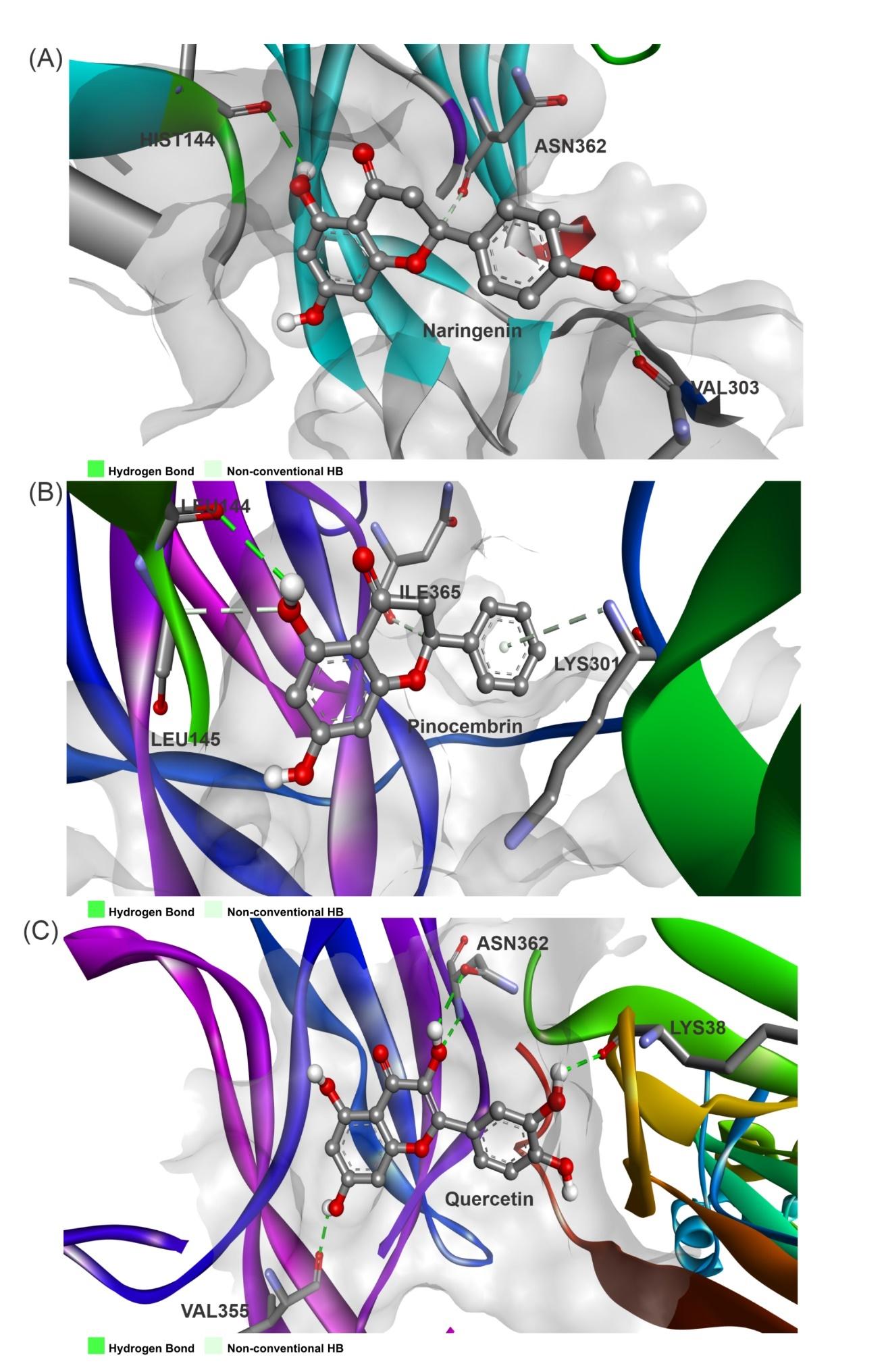


**Figure S7:** Key binding interactions of top-ranked flavonoids with zikv envelope protein after qm/mm optimization. (A) Naringenin forming conventional hydrogen bonds with HIS144 and ASN362, and additional interactions with VAL303; (B) Pinocembrin stabilized by hydrogen bonding with LEU145 and HIS144, and non-conventional interactions with ILE365 and LYS301; (C) Quercetin establishing multiple hydrogen bonds with ASN362, VAL355, and LYS38, highlighting a dense interaction network within the binding pocket. Hydrogen bonds are shown in green; non-conventional H-bonds in light green.
